# The effect of donation activity dwarfs the effect of lifestyle, diet and targeted iron supplementation on blood donor iron stores

**DOI:** 10.1371/journal.pone.0220862

**Published:** 2019-08-13

**Authors:** Muriel Lobier, Johanna Castrén, Pia Niittymäki, Elina Palokangas, Jukka Partanen, Mikko Arvas

**Affiliations:** Research and Development, Finnish Red Cross Blood Service, Helsinki, Finland; Pennsylvania State University, UNITED STATES

## Abstract

The iron status of blood donors is a subject of concern for blood establishments. The Finnish Red Cross Blood Service addresses iron loss in blood donors by proposing systematic iron supplementation for demographic at-risk donor groups. We measured blood count, ferritin and soluble transferrin receptor (sTfR) and acquired lifestyle and health information from 2200 blood donors of the FinDonor 10000 cohort. We used modern data analysis methods to estimate iron status and factors affecting it with a special focus on the effects of the blood service’s iron supplementation policy. Low ferritin (< 15 μg/L), an indicator of low iron stores, was present in 20.6% of pre-menopausal women, 10.6% of post-menopausal women and 6% of men. Anemia co-occurred with iron deficiency more frequently in pre-menopausal women (21 out of 25 cases) than in men (3/6) or post-menopausal women (1/2). In multivariable regression analyses, lifestyle, dietary, and blood donation factors explained up to 38% of the variance in ferritin levels but only ~10% of the variance in sTfR levels. Days since previous donation were positively associated with ferritin levels in all groups while the number of donations during the past 2 years was negatively associated with ferritin levels in pre-menopausal women and men. FRCBS-provided iron supplementation was negatively associated with ferritin levels in men only. Relative importance analyses showed that donation activity accounted for most of the explained variance in ferritin levels while iron supplementation explained less than 1%. Variation in ferritin levels was not significantly associated with variation in self-reported health. Donation activity was the most important factor affecting blood donor iron levels, far ahead of e.g. red-meat consumption or iron supplementation. Importantly, self-reported health of donors with lower iron stores was not lower than self-reported health of donors with higher iron stores.

## Introduction

Recent studies have indicated that blood donation may be associated with iron depletion but only a few studies have systematically scrutinized which donor-related factors predominantly explain this depletion [1–5]. Iron deficiency, if left untreated, leads to iron deficient erythropoiesis, and, finally, to anemia [6]. While anemia or low hemoglobin (Hb) can be detected with point-of-care tests [7], these tests cannot detect iron-deficiency in donors who still maintain normal Hb levels. Policies used by blood establishments to minimize the risk of iron deficiency include minimum inter-donation intervals, ferritin screening, and targeted iron supplementation [8–13]. Iron supplementation has been shown to improve and accelerate the replacement of iron stores in blood donors [14–18], but there is currently no retrospective study specifically investigating the population-level effect of targeted iron supplementation for at-risk groups.

There are only a few blood donation establishments who have implemented a systematic population-level supplementation policy to manage iron deficiency in blood donors [8,10,19,20]. In Denmark, guided iron supplementation is provided to at-risk donors identified through a Hb and ferritin screening program [9,19]. The Finnish Red Cross Blood Service (FRCBS) has implemented since the 1980s an iron supplementation policy targeted towards at-risk donor groups determined as a function of demographics and donation activity. Iron tablets are provided at donation time to all female donors under the age of 50 and to all other donors whose preceding donation was less than 4 months ago. While previous retrospective studies have reported on the effect of iron supplementation on iron stores of blood donors with contrasted results [1,5], they did not directly address the effect of a systematic iron supplementation policy. Investigating donor iron status in Finland can provide novel insights on the population-level effect of a large-scale targeted iron supplementation.

Iron levels are affected not only by blood donation activity but also by other physiological, lifestyle, and dietary factors. Age [21], BMI [21–23], alcohol [22] and meat consumption [21,24] have been positively associated with iron stores [1], whereas dairy products [25,26] and menstruation[25] have been negatively associated with iron stores both in the general population and in blood donors[1]. However, to determine the practical relevance for blood donor iron stores of targeted iron supplementation and lifestyle and dietary recommendations, deeper data and statistical analyses are needed. In the present study, we estimate the amount of inter-individual variance in iron levels explained by blood donation activity as well as by dietary and lifestyle factors, and can therefore compare their impact on a blood donor population.

The health effects of iron deficiency without anemia are still debated[27,28]. In previous blood donor research, ferritin levels did not affect self-reported health[29] or the presence of restless leg syndrome [30]. However, decreased ferritin levels have been associated with the presence of undesirable side-effects of donation [11]. In the present study, we used ferritin and sTfR to investigate the iron status of Finnish blood donors, to quantify the effect of targeted iron supplementation on iron levels across these individuals, and to assess the influence of donor iron levels on self-reported health.

## Methods

### Study population and data collection

FinDonor 10 000 is a prospective longitudinal cohort study initiated by the FRCBS. An ethical approval was obtained from the Helsinki and Uusimaa Hospital District Review Board (approval number 282/13/03/00/14). Participants gave written informed consent before the beginning of the standard blood donation procedure. The study ran from May 18^th^ 2015 to December 8^th^ 2017. All prospective whole-blood donors (ages 18–70) in the three donation sites located in the Helsinki area were invited to participate in the study. Data were collected from 2584 whole-blood donors. Details of the FinDonor cohort, sample collection, laboratory analyses, and quality issues are described elsewhere [31]. Blood samples were collected when donors visited donation centers for whole-blood donations regardless of whether the donation attempt succeeded. Permanent deferral was the only exclusion criteria. Donors with low hemoglobin (Hb < 125 g/L for women and < 135 g/L for men) were deferred from donation as per FRCBS policy. For successful donations, samples were collected from the diversion pouch at the beginning of the donation. For deferred donations, separate venous samples were drawn. The blood count, C-reactive protein (CRP), ferritin, and soluble transferrin receptor (sTfR) were measured. Participants answered an electronic questionnaire at the donation site during their enrollment visit. This questionnaire included 28 questions about general health, current and past medical conditions, dietary habits, smoking, alcohol use, physical activity, and sleep. Once enrolled, donors could provide blood samples at each one of their whole-blood donation attempt in a participating donation center. For the purposes of this specific study, we analyzed data from donors’ first study blood sample.

Participants were offered iron supplementation according to the standard operating procedure of the FRCBS. Blood donation staff is instructed to offer iron supplementation to 1) all women under 50 years old at all donations and to 2) all other donors donating every 4 months or more frequently. Donors were primarily offered 20 daily tablets containing 25 mg of ferrous fumarate and 350 mg of animal hemoglobin, or alternatively 30 daily tablets containing 100 mg of ferrous sulfate. Participants were asked in the electronic questionnaire if they had been offered iron supplementation at their previous donation and, if they had, how much of the offered iron they had ingested. Donors were queried in separate questions regarding their self-administered nutritional supplement intake (iron, vitamin C, multivitamins).

### Statistical analyses

Statistical analyses were performed using R statistical software[32]. Analysis code is available at https://github.com/FRCBS/iron_levels_of_blood_donors. Data will be made available to individual researchers upon reasonable request. Population characteristics were described by mean and standard deviation (SD) for normally distributed continuous variables, median and interquantile range (IQR) for non-normally distributed variables and as the percentage of the frequency distribution for each category for categorical variables. We performed pair-wise comparisons of population characteristics between men, pre-menopausal and post-menopausal women using two-sample t-tests for normally distributed variables, Wilcoxon rank sum tests for non-normally distributed variables and chi-squared tests for categorical variables. We report the adjusted p-values after Holm-Bonferroni correction for multiple comparisons for these comparisons and use an alpha-level of 0.05 to identify significant comparisons.

We determined the prevalence of iron deficiency (ID), low hemoglobin and anemia in each donor groups. ID was defined either as ferritin < 15 μg/L[33] (ID_Ferritin_) or as sTfR ≥ 4.4 mg/L for women or ≥ 5.0 mg/L for men (ID_sTfR_)[34]. Low hemoglobin was defined according to FRCBS low hemoglobin deferral rules, i.e. Hb < 125 g/L for women and < 135 g/L for men. Anemia was defined according to WHO standards as Hb <120 g/L for women and <130 g/L for men.

### Multivariable analyses of ferritin and sTfR levels

We investigated predictors of ferritin and sTfR levels using multiple ordinary least squares (OLS) linear regression and robust linear regression analyses. Robust regression [35,36] is an alternative to OLS regression when data is contaminated with overly influential observations. Robust regression coefficient estimates are less affected by the presence of influential observations. Robust regression and associated p-values were computed using the MASS[37] and the sfsmic[38] packages respectively. We used bootstrapping (boot package[39]) to determine the 95% Bias Corrected and Accelerated Confidence Intervals[40] (BC_a_ CIs) for regression coefficients.

Covariates related to blood donation history were the number of days since the last donation and the number of donations in the last two years. To account for the non-linear relationship between ferritin levels and the number of donations in the last two years in at least one stratified group, a quadratic term was added. Continuous covariates related to physiologic factors were age, BMI, and CRP. Ferritin and CRP were log-transformed while the number of days since last donation was log transformed and divided by log(2) to facilitate coefficient interpretation. Binary covariates were smoking status (Yes/No), and, for women, nulliparous status (Yes/No).

Iron supplementation, dietary factors and alcohol consumption were also entered in the model. As one of the main goals of this study was to assess the efficiency of the FRCBS iron supplementation policy, we only took under consideration reported intake of FRCBS-provided iron supplementation. Iron supplementation was recoded to a 1 to 4 numeric scale with 1 corresponding to no FRCBS supplementary iron intake (not offered or not taken), 2 to limited compliance (less than half the provided course taken), 3 to partial compliance (at least half the provided course taken) and 4 to full compliance (all or almost all the provided course taken). Consumption frequency for dietary factors was recoded to a numeric scale from 1 (never) to 4 (daily or more). Iron supplementation, dietary factors and alcohol consumption were entered in the regression models as continuous variables[41].

We quantified the relative importance[42], i.e. the proportionate contribution of each covariate to the total model R^2^ taking into account both its direct effect and its effect when combined with other covariates, in predicting ferritin and sTfR levels for covariates that were consistently significant for both OLS and robust regressions in at least one donor group. We used PMVD as a measure of relative importance and used the relaimpo package[42] to compute it and its bootstrapped 95% BC_a_ CIs.

### Multivariable analyses of self-reported health

Self-reported health was assessed using the question “How would you rate your recent health in general” to which participants answered on a 5 point scale ranging from poor to excellent. This very general, seemingly subjective, simple question is one of the most commonly used in health interview surveys as a public health indicator[43]. We used multivariable ordinal logistic models to test the association of ferritin and self-reported health in men and women; pre-menopausal and post-menopausal women were collapsed to improve statistical power. We adjusted for age, BMI, CRP, number of donations in the last two years, number of days since the last donation and physical activity (both groups) and menopausal status (women). We log transformed the ferritin values and further divided it by log(2) to facilitate interpretation of regression coefficients. We used the rms[44] package to run ordinal logistic regression analyses and the brms[45] package to fit Bayes ordinal models. We report odds-ratio and their bootstrapped 95% Bca confidence intervals for ordinal logistic regression and odds-ratios and their 95% highest density intervals (HDI) for Bayesian ordinal models. In addition, we evaluated whether a model including ferritin had a better predictive accuracy (defined as elpd, expected log pointwise predictive density) of donor self-reported health than a model without ferritin using leave-one-out cross-validation[46]. Better predictive accuracy can be inferred when the differences between model elpds are positive and orders of magnitude larger than the standard error.

## Results

A total of 2584 donors enrolled the study and the data collected during the first study visit of each donor were used. We excluded from data analyses first-time donors (N = 41), donors missing ferritin, sTfR or CRP measurements (N = 7), and donors who did not answer to the items of the questionnaire included in the present data analysis (N = 245). Women of 45 years of age or older reporting amenorrhea formed the post-menopausal group. Women under 45 years of age reporting amenorrhea were excluded from all analyses (N = 81). Women of all ages reporting the presence of menstruation formed the pre-menopausal group. In addition, we removed from all analyses donors (N = 10) with extreme physiologic outliers (BMI > 50, Ferritin > 400 and CRP > 20).

Complete data were available from 2200 donors: 846 pre-menopausal women, 452 post-menopausal women and 902 men. Characteristics of the study population are presented in Table 1. 65% of pre-menopausal women, 43% of post-menopausal women and 35% of men reported being provided with iron at their previous blood donation visit (S1 Fig). Smoking frequency and milk consumption were the only variables with no between-group differences, for all other variables at least one significant difference was found.

**Table 1:** Basic characteristics of study groups.

	Pre-menopausal women	Post-menopausal women	Men	(1) vs (2)	(1) vs (3)	(2) vs (3)
N	846	452	902			
age*	34 (10)	58 (6)	45 (14)	p < .0001	p < .0001	p < .0001
BMI **	24.2 (22.1, 28.1)	25.2 (23.0, 28.3)	25.7 (23.9, 28.1)	p < .05	p < .0001	n.s.
Smoking (yes)	13.6%	9.3%	10.5%	n.s.	n.s.	n.s.
Pregnancy = yes (%)	30%	77%		p < .0001		
Hemoglobin*	135.5 (8.4)	138.2 (8.6)	150.6 (9.4)	p < .0001	p < .0001	p < .0001
Ferritin **	26 (16, 41)	34 (22, 52)	42 (25, 68)	p < .0001	p < .0001	p < .0001
sTfR **	3.3 (2.7, 4.2)	3.1 (2.5, 3.8)	3.4 (2.7, 4.1)	n.s.	p < .0001	p < .01
CRP **	2.9 (2.9, 2.9)	2.9 (2.9, 2.9)	2.9 (2.9, 2.9)	p < .01	n.s.	p < .001
Number of donations (2 years)*	2.6 (1.7)	3.5 (1.9)	4.6 (2.7)	p < .0001	p < .0001	p < .0001
Time to previous donation (days) **	177 (112, 325)	140 (104, 241)	111 (78, 203)	p < .001	p < .0001	p < .0001
Iron supplementation ƚ	42, 12, 9, 38	62, 5, 5, 28	67, 6, 4, 22	p < .0001	p < .0001	n.s.
Red meat ǂ	16, 15, 58, 12	8, 25, 63, 4	5, 10, 71, 15	p < .0001	p < .0001	p < .0001
Vegetables ǂ	0, 1, 26, 73	0, 1, 19, 80	0.1, 2, 43, 55	n.s.	p < .0001	p < .0001
Fruit and berries ǂ	0, 4, 37, 59	0.2, 2, 24, 74	0.3, 8, 48, 44	p < .0001	p < .0001	p < .0001
Milk ǂ	5, 6, 21, 68	5, 6, 18, 72	5, 7, 23, 66	n.s.	n.s.	n.s.
Fruit juices ǂ	14, 48, 28, 10	22, 40, 22, 16	9, 34, 36, 21	p < .001	p < .0001	p < .0001
Coffee ǂ	17, 5, 8, 70	9, 2, 5, 85	13, 4, 7, 76	p < .0001	n.s.	p < .05
Tea ǂ	13, 29, 30, 28	14, 27, 23, 37	19, 33, 24, 24	n.s.	p < .001	p < .0001
Beer ǂǂ	61, 32, 7, 0.2	64, 27, 9, 0.4	28, 37, 31, 4	n.s.	p < .0001	p < .0001
Wine ǂǂ	32, 55, 13, 0.4	30, 45, 23, 2	38, 46, 14, 1	p < .0001	p < .01	p < .01
Liquor ǂǂ	83, 16, 0.4, 0.1	92, 7, 1, 0	65, 31, 5, 0.1	p < .001	p < .0001	p < .0001
Daily physical activity ǂǂǂ	3, 19, 48, 30	4, 13, 41, 43	6, 21, 42, 31	p < .001	n.s.	p < .001
Exercise frequency ǂǂǂǂ	7, 8, 18, 67	5, 6, 17, 72	8, 9, 22, 61	n.s.	n.s.	p < .05

* Normally distributed variable are summarized as mean (SD).

** Non-normally distributed variable are summarized as median (25th, 75th percentile).

ƚ Shown as percentage of the frequency distribution of the categories: 1 (“Not provided or none ingested”, 2 (“Less than half of course ingested”), 3 (“At least half of course ingested”, 4 (All of nearly all the course ingested”).

ǂ Shown as percentage of the frequency distribution of the categories: 1 (“Never”), 2 (“Less than once weekly”), 3 (“From 1 to 6 times per week”), 4 (“Daily or more often”)

ǂǂ Shown as percentage of the frequency distribution of the categories: 1 (“Rarely or never”), 2 (“A few per month”), 3 (“A few per week”), 4 (“Daily or almost”)

ǂǂǂ Shown as percentage of the frequency distribution of the categories: 1 (“Less than 15 min”), 2 (“15 to 30 min”), 3 (“30 to 60 min”), 4 (“Longer than one hour”)

ǂǂǂǂ Shown as percentage of the frequency distribution of the categories: 1(“Less than monthly”), 2 (“Once to twice monthly”), 3 (“Once weekly”), 4 (“Twice weekly or more often”)

Group differences are tested with an independent two-sample t-test for normally distributed variables, with a Mann-Whitney test for non-normally distributed variables and chi-squared test for categorical variables.

### Prevalence of iron deficiency, low hemoglobin and anemia in Finnish blood donors

**Fig 1:**
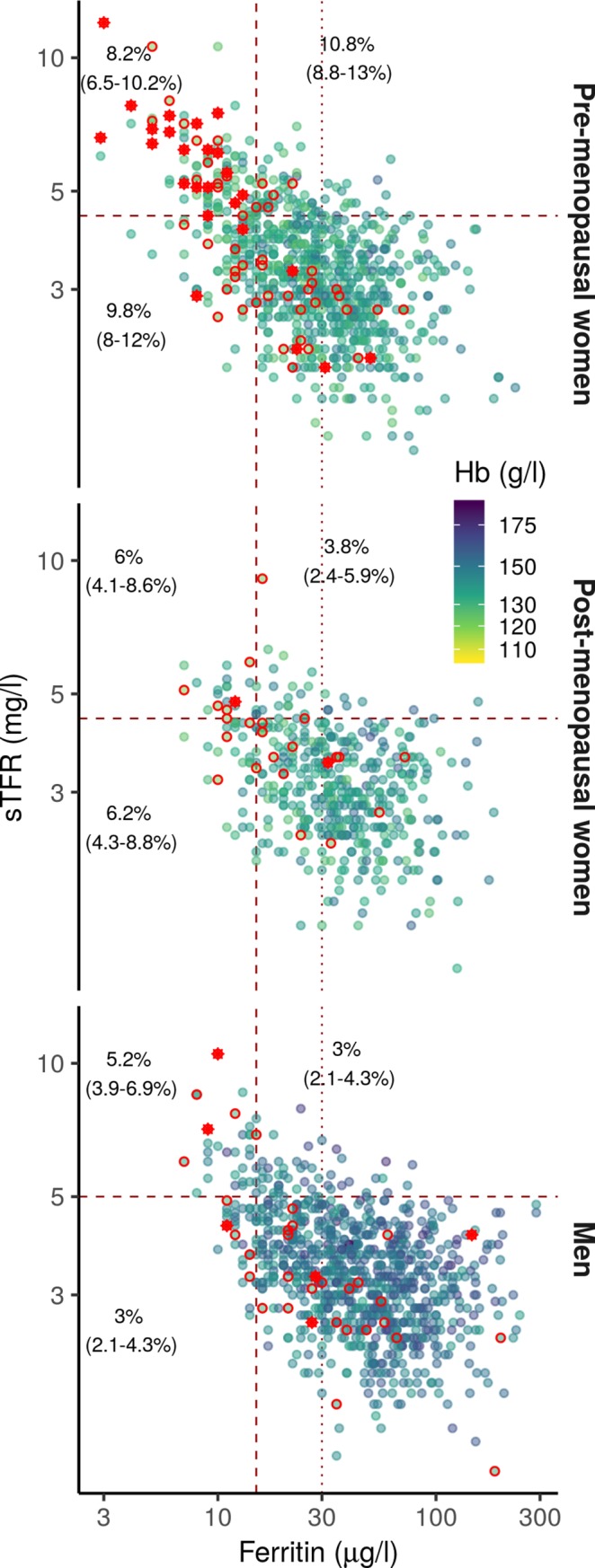
Scatterplots of sTFR concentration as a function of ferritin concentration show that anemic donors are not systematically iron deficient.

The majority of blood donors had ferritin and sTfR values in the normal range (Fig 1). We determined the percentage of ID, with ID defined as ferritin < 15 μg/l and/or sTfR > 4.4 mg/L for women and > 5 mg/L for men (Table 2). Iron deficiency was most prevalent in pre-menopausal women, followed by post-menopausal women and men. Only 5 to 8% of donors had both low ferritin and high sTfR (Fig 1). We determined the percentage of blood donors with anemia as defined by the WHO (Venous Hb < 120 g/l for women and < 130 g/L for men) and low hemoglobin as defined by low hemoglobin deferral thresholds (Venous Hb < 125 g/l for women and < 135 g/L for men). Anemia and low hemoglobin were, similar to iron deficiency, most prevalent in pre-menopausal women, then post-menopausal women and then men. Iron deficiency (low ferritin and/or high sTfR) was present in 84% (21 of 25), 50% (1 of 2) and 50% (3 of 6) of anemic pre-menopausal women, post-menopausal women, and men, respectively. Furthermore, iron deficiency was present in 66.2% (47 of 71), 41.7% (10 of 24) and 31.4% (11 of 35) of low hemoglobin pre-menopausal women, post-menopausal women, and men, respectively.

**Table 2:** Percentage of donors with abnormal hemoglobin, ferritin or sTfR values.

	Pre-menopausal women	Post-menopausal women	Men
Anemia	3 (2–4.3)	0.4 (0.1–1.6)	0.7 (0.3–1.4)
Low hemoglobin	8.4 (6.7–10.5)	5.3 (3.6–7.8)	3.9 (2.8–5.3)
Low ferritin	20.6 (18–23.4)	10 (7.5–13.1)	6 (4.6–7.7)
High sTfR	18.9 (16.4–21.7)	9.7 (7.3–12.8)	8.2 (6.6–10.2)

Text in quadrants indicates the percentage of donors with isolated low ferritin (bottom left), isolated high sTfR (top right) and concurrent low ferritin and high sTfR (top left). Color represents hemoglobin concentration. Red circles highlight donors with low hemoglobin. Red asterisks represent anemic donors.

### Determinants of iron status in Finnish blood donors

**Fig 2:**
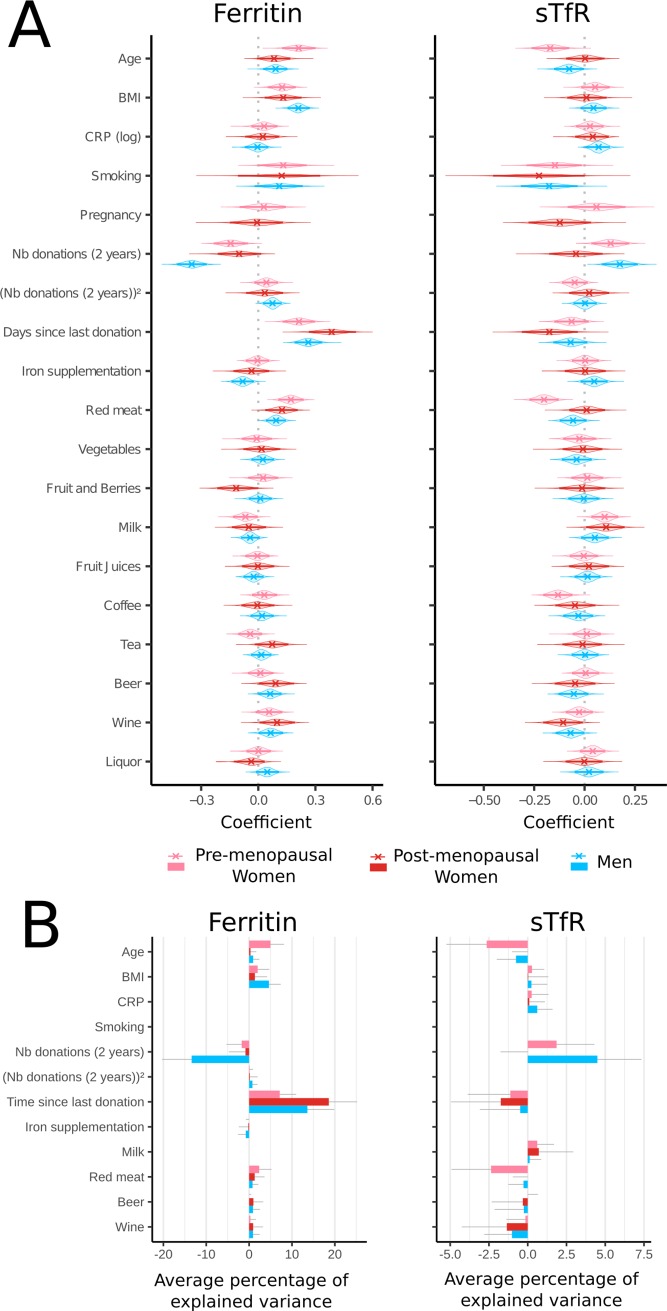
Donation activity is the most consistent explanatory variable of ferritin levels but explains limited variance in sTfR levels. (A) The standardized coefficients (cross), their 95% BCa confidence intervals (thick line) and bootstrap distributions are plotted for all covariates entered in the robust regressions. (B) The relative importance (average percentage of variance in the data explained by the co-variate) for co-variates consistently significant in at least one group are plotted as positive values if there was a positive correlation between the outcome variable and the co-variate and as negative values if there was a negative correlation between the outcome variable and the co-variate. Grey lines represent bootstrapped 95% CIs.

Multivariable linear regression analyses were carried out to identify factors associated with variation in iron status as measured by serum ferritin and sTfR. Analyses were run separately for pre-menopausal women, post-menopausal women and men. As influential observations were present (S2 and S3 Figs), we carried out both Ordinary Least Squares (OLS) (S1 and S2 Tables) and robust regression (Tables 3 and 4). We report both regression coefficients (Tables) and standardized coefficients (Fig 2). Results were qualitatively similar for both model types for most covariates (Fig 2A).

**Table 3:** Multiple robust regression results for ferritin levels.

	Pre-menopausal women		Post-menopausal women		Men	
	Coefficient (95% CI)	p-values	Coefficient (95% CI)	p-values	Coefficient (95% CI)	p-values
Age	0.07 (0.04, 0.10)	<0.0001	0.05 (-0.003, 0.10)	0.072	0.02 (0.01, 0.04)	0.004
BMI	0.02 (0.01, 0.03)	0.0005	0.02 (0.005, 0.03)	0.006	0.04 (0.03, 0.05)	<0.0001
CRP	0.06 (-0.08, 0.21)	0.367	0.06 (-0.18, 0.29)	0.601	-0.02 (-0.26, 0.19)	0.883
Smoking(yes)	0.09 (0.001, 0.18)	0.047	0.08 (-0.06, 0.20)	0.273	0.08 (-0.01, 0.16)	0.080
Pregnancy(Yes)	0.02 (-0.06, 0.10)	0.649	-0.004 (-0.09, 0.08)	0.929		
Nb donations (2 years)	-0.06 (-0.10, -0.02)	0.002	-0.03 (-0.07, 0.01)	0.105	-0.09 (-0.11, -0.07)	<0.0001
(Nb donations (2 years))^2^	0.01 (-0.005, 0.03)	0.165	0.01 (-0.01, 0.02)	0.450	0.01 (0.002, 0.01)	0.004
Time since last donation (days)	0.13 (0.08, 0.19)	<0.0001	0.23 (0.16, 0.31)	<0.0001	0.17 (0.12, 0.21)	<0.0001
Iron supplementation	-0.002 (-0.03, 0.03)	0.895	-0.02 (-0.06, 0.03)	0.450	-0.04 (-0.08, -0.01)	0.005
Red meat	0.13 (0.08, 0.19)	<0.0001	0.11 (0.03, 0.19)	0.006	0.10 (0.04, 0.16)	0.001
Vegetables	-0.01 (-0.13, 0.10)	0.822	0.03 (-0.11, 0.17)	0.735	0.03 (-0.05, 0.10)	0.433
Fruit and Berries	0.03 (-0.07, 0.13)	0.505	-0.14 (-0.27, 0.001)	0.034	0.01 (-0.05, 0.08)	0.737
Milk	-0.06 (-0.11, -0.004)	0.038	-0.04 (-0.11, 0.03)	0.269	-0.04 (-0.08, 0.01)	0.120
Fruit Juices	-0.004 (-0.06, 0.05)	0.889	-0.001 (-0.06, 0.05)	0.959	-0.02 (-0.06, 0.02)	0.373
Coffee	0.02 (-0.02, 0.06)	0.347	-0.003 (-0.07, 0.06)	0.915	0.01 (-0.03, 0.05)	0.484
Tea	-0.03 (-0.07, 0.01)	0.185	0.04 (-0.01, 0.09)	0.112	0.01 (-0.02, 0.04)	0.563
Beer	0.01 (-0.06, 0.08)	0.740	0.08 (-0.0001, 0.17)	0.046	0.05 (0.0004, 0.10)	0.044
Wine	0.06 (-0.01, 0.14)	0.098	0.08 (-0.001, 0.16)	0.034	0.06 (0.003, 0.12)	0.040
Liquor	0.001 (-0.11, 0.11)	0.983	-0.07 (-0.24, 0.07)	0.410	0.06 (-0.02, 0.13)	0.113

**Table 4:** Multiple robust regression results for sTfR levels.

	Pre-menopausal women		Post-menopausal women		Men	
	Coefficient (95% CI)	p-values	Coefficient (95% CI)	p-values	Coefficient (95% CI)	p-values
Age	-0.03 (-0.04, -0.01)	0.0003	0.001 (-0.02, 0.03)	0.958	-0.01 (-0.02, -0.0002)	0.043
BMI	0.003 (-0.001, 0.01)	0.165	0.001 (-0.01, 0.01)	0.853	0.003 (-0.002, 0.01)	0.205
CRP	0.03 (-0.04, 0.09)	0.460	0.05 (-0.06, 0.14)	0.413	0.11 (0.01, 0.20)	0.041
Smoking(yes)	-0.05 (-0.09, -0.003)	0.040	-0.07 (-0.13, 0.001)	0.068	-0.05 (-0.10, -0.01)	0.023
Pregnancy(Yes)	0.02 (-0.03, 0.07)	0.389	-0.04 (-0.08, 0.01)	0.132		
Nb donations (2 years)	0.03 (0.01, 0.04)	0.009	-0.01 (-0.03, 0.01)	0.533	0.02 (0.01, 0.03)	0.0003
(Nb donations (2 years))^2^	-0.01 (-0.01, 0.002)	0.138	0.002 (-0.01, 0.01)	0.641	0.0001 (-0.002, 0.003)	0.938
Time since last donation (days)	-0.02 (-0.04, 0.01)	0.164	-0.05 (-0.09, -0.01)	0.015	-0.02 (-0.04, 0.003)	0.104
Iron supplementation	0.001 (-0.02, 0.02)	0.934	0.001 (-0.02, 0.02)	0.957	0.01 (-0.005, 0.03)	0.172
Red meat	-0.08 (-0.10, -0.05)	< 0.0001	0.004 (-0.03, 0.04)	0.834	-0.03 (-0.06, 0.005)	0.090
Vegetables	-0.02 (-0.08, 0.04)	0.527	-0.01 (-0.07, 0.06)	0.890	-0.02 (-0.06, 0.02)	0.300
Fruit and Berries	0.01 (-0.04, 0.05)	0.735	-0.01 (-0.07, 0.06)	0.838	-0.002 (-0.04, 0.03)	0.926
Milk	0.04 (0.01, 0.07)	0.005	0.04 (0.003, 0.07)	0.032	0.02 (-0.01, 0.05)	0.130
Fruit Juices	-0.002 (-0.03, 0.03)	0.909	0.01 (-0.02, 0.04)	0.665	0.01 (-0.02, 0.03)	0.641
Coffee	-0.04 (-0.06, -0.02)	0.0003	-0.02 (-0.05, 0.02)	0.342	-0.01 (-0.03, 0.01)	0.372
Tea	0.004 (-0.02, 0.03)	0.741	-0.003 (-0.03, 0.02)	0.848	0.001 (-0.02, 0.02)	0.934
Beer	0.003 (-0.03, 0.04)	0.881	-0.02 (-0.06, 0.02)	0.347	-0.02 (-0.05, 0.01)	0.159
Wine	-0.01 (-0.05, 0.02)	0.470	-0.04 (-0.08, -0.004)	0.036	-0.03 (-0.06, 0.0001)	0.071
Liquor	0.03 (-0.02, 0.09)	0.248	-0.001 (-0.08, 0.08)	0.984	0.01 (-0.03, 0.05)	0.540

**Fig 3:**
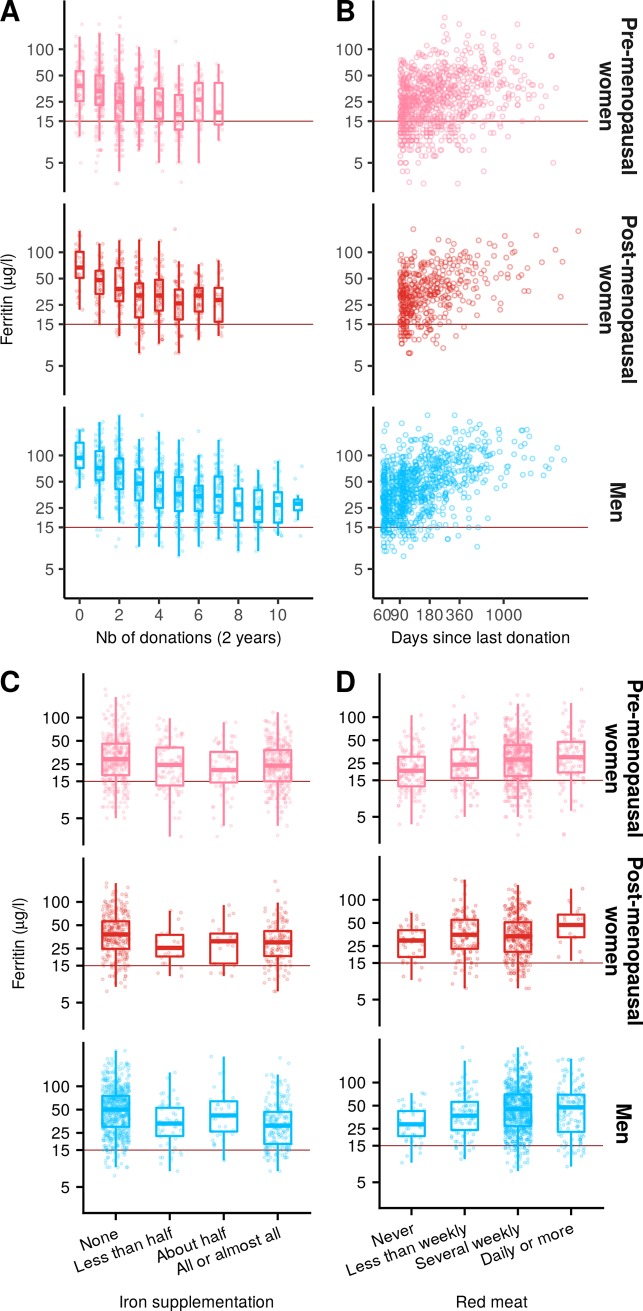
Ferritin levels are more affected by donation history than by iron intake. (A) Ferritin levels are plotted as a function of number of donation in the last two years. (B) Ferritin levels are plotted as a function of days since last donation. (C) Ferritin levels are plotted as a function of iron supplementation. (D) Ferritin levels are plotted as a function of red meat consumption. Boxplots are superimposed for each number of donations, iron supplementation level and red-meat consumption level.

The amount of variance in iron levels explained by the chosen factors varied not only between ferritin and sTfR but also amongst the three groups (Fig 2B). Models with sTfR as an outcome variable explained only a limited amount of variance (pre-menopausal women: R^2^ = 0.12, post-menopausal women: R^2^ = 0.06 and men: R^2^ = 0.10). The overall model was significant solely for pre-menopausal women and men (S2 Table). STfR could not be well predicted by the available covariates (S3 Fig). We therefore only report extensively the results for ferritin for which the amounts of variance explained by the models were consistent with small to medium effect sizes (S1 Table). Fig 3 shows the relationship between ferritin and sTfR respectively for factors related to blood donation activity (number of donations in the last two years and Time since last donation) and to iron intake (red meat consumption and iron supplementation) as these factors relate directly to FRCBS policies and/or recommendations made to donors. The relationship between ferritin and other factors are presented in S5 Fig and S6 Fig for factors significant in at least 2 demographic groups.

Only some of the factors significantly co-varied with ferritin levels and many of them were significant only in one or two of the three study groups. Increased age and BMI were consistently associated with increased ferritin levels. For post-menopausal women, age was a significant co-variate only for the OLS model, suggesting that the effect in that group could be driven by a few influential observations. Smoking was significantly positively correlated with ferritin levels for pre-menopausal women only.

The factors related to blood donation affected ferritin levels differently between the three study groups. The number of donations in the last two years was negatively associated with ferritin levels only in pre-menopausal women and men, with a 5.7% and 8.5% decrease, respectively, in ferritin levels for each additional donation (Fig 3A). The squared number of donations in the last two years was positively associated with ferritin levels only in men. The combination of a negative correlation for the covariate and a positive correlation for the squared covariate indicates that the negative effect of increased blood donation activity on ferritin levels was larger for donors who donated rarely than for donors who donated frequently. Doubling the number of days since the previous donation was associated with 14%, 26% and 18% increases in ferritin levels for pre-menopausal women, post-menopausal women and men, respectively (Fig 3B).

Iron supplementation had a significant influence on ferritin levels only in men (Fig 3C): an increase in iron supplementation was associated with lower ferritin levels. On average, men who reported taking at least half of the iron supplementation had ferritin levels 4% lower than those who reported not being offered or not taking the offered supplementation. The pattern of results for iron supplementation was similar both qualitatively and quantitatively when analyses were run with both FRCBS-provided and self-administered iron supplementation were collapsed into a binary (no iron / at least some iron) predictor (data not shown).

The majority of dietary factors did not have any effect on ferritin levels. The only effect consistently significant across all three groups was a positive association between red meat consumption and ferritin levels (Fig 3D). Increased milk consumption was associated with decreased ferritin levels in pre-menopausal women only. Beer and wine consumptions were associated with increased ferritin levels in men and post-menopausal women.

To assess the relative importance of each covariate in explaining variation in ferritin levels, we computed the relative importance of the covariates significant in at least one study group (Fig 2B). For pre-menopausal women, the number of days since previous donation and age had the largest relative importance, with lower relative importance values for red meat and BMI. For post-menopausal women, the number of days since previous donation was the only covariate with a relative importance above 2%. For men, the number of days since the previous donation and the number of donations in the previous two years had similar relative importance, with lower relative importance for BMI. Overall, donation activity was the main source of variability in ferritin levels in our models. Iron supplementation explained less than one percent of variance for all groups.

### Ferritin levels and donor self-reported health

**Fig 4:**
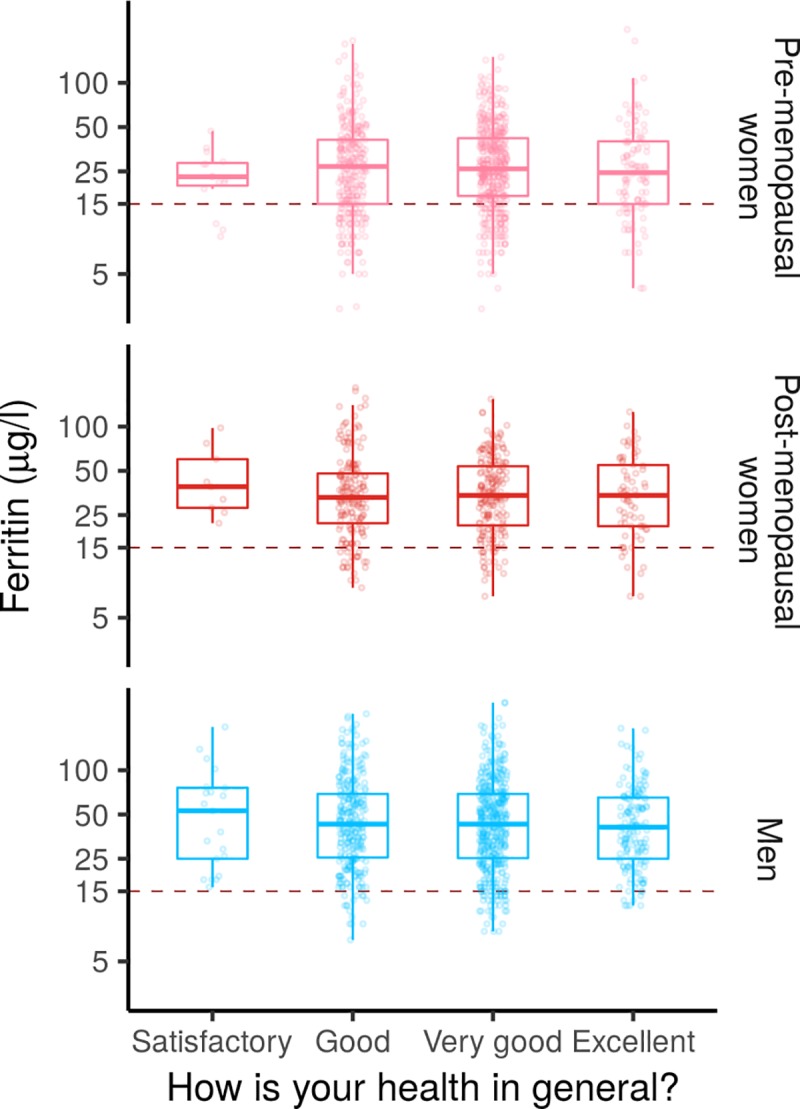
Self-reported health is not affected by donor iron levels. Donor ferritin levels are plotted as a function of their self-reported health status and standard boxplots are superimposed for each self-reported health status.

We investigated whether ferritin levels co-varied with donor self-reported health using ordinal logistic regression (Fig 4). To improve statistical power, we combined the pre-menopausal and post-menopausal women into a single group. Almost all donors reported their health to be at least good (S4 Fig). When age, BMI, CRP, smoking, menopausal status, blood donation activity and physical activity were controlled for, a doubling of ferritin values increased the odds of reporting one’s health as very good or excellent compared to satisfactory or good by 11% (OR = 1.11, 95%CI .98–1.25) for women and 6% for men (OR = 1.06, 95%CI .91–1.24), but neither of these increases were statistically significant (S3 Table). The difference in predictive accuracy between Bayesian models with and without ferritin as a covariate was of the same magnitude as its standard error, suggesting that ferritin did not improve the predictive accuracy of the model (women: difference = -0.7, SE = 3.5, men: difference: difference = 1.3, SE = 1.5).

## Discussion

The increased risk for iron deficiency in regular blood donors is well documented [2–4,9,47], but the importance of the different factors affecting iron stores and in particular, population-level effects of systematic iron supplementation of at-risk donor groups on iron levels and health are poorly understood. In the present study, we addressed these questions in the Finnish blood donor study cohort by using modern data analysis tools. Our three major findings were the following. (i) First, iron deficiency was present in the Finnish donor population at levels similar to other donor populations despite iron supplementation. However, it is of note that hemoglobin levels, the current standard for donor health screening in blood banks, correlated poorly with iron deficiency. Most low hemoglobin men were not ID, suggesting that their low hemoglobin was not directly, or perhaps at all, related to their iron status. (ii) Second, the effect of iron supplementation on donor iron levels was negligible compared to the effect of donation activity, i.e. time from previous donation and number of donations during the previous 2 years. (iii) Finally, there was no evidence of harmful effects of blood donation-related iron depletion on self-reported health.

Our donor population had lower prevalence of anemia but somewhat higher prevalence of low hemoglobin than comparable populations. Anemia was present in only 3%, 0.4% and 0.7% of pre-menopausal women, post-menopausal women and men, respectively. While these low rates could appear surprising given the WHO’s predicted European anemia rates of 19.9% (95%CI 13.5–28.4) for pre-menopausal women [48] or recent estimations of the prevalence of anemia in Portugal [49] (20.7 in pre-menopausal women, 18.9% in men and around 20% in older women), they are consistent with previous estimations in the Finnish population in the late 1990s [50]. Prevalence was then 5.9% in women under 50 years of age, 5.5% in women over 50 years of age and 1.3% in men. The lower prevalence observed in our data could results from the inherent bias of a blood donor population: new donors with low hemoglobin and/or anemia at their first visit usually do not enter the donor pool. These donors would be de facto excluded from analysis since our sample for this study includes only repeat donors. Overall, very low anemia rates show that FRCBS policies successfully mitigate anemia in blood donors. Low hemoglobin was present in 8.4%, 5.3% and 3.9% of pre-menopausal women, post-menopausal women and men, respectively. These rates are higher than those in the Capital region of Denmark (less than 1% for men and less than 4% for women) [19] and are higher than most reported deferral rates for countries using the same thresholds [13]. This discrepancy can be explained by (1) different screening policies and (2) poor sensitivity of point-of-care fingerpick Hemocue measurements compared to venous sample analysis in detecting low Hb. Recomputing low Hb rates with the point-of-care screening Hb value instead of the venous sample Hb value results in low Hb rates (6.6%, 2.4% and 2.2% for pre-menopausal women, post-menopausal women and men, respectively) which are more consistent with reports from other countries [13]. Furthermore, our low venous Hb rates are similar to or lower than low Hb rates from venous samples in the INTERVAL study (around 5.3 to 5.5% in man and 13.4 to 13.9% in women). Overall these results show that the current FRCBS screening strategy reasonably ensures that most anemic and low hemoglobin blood donors are excluded from the donor pool.

Iron deficiency was present in the donor population in proportions smaller than but still comparable to other studies [10]. If taking only into consideration ID as defined by ferritin levels below 15 μg/L (hereafter ID_ferr_), the prevalence in our population of post-menopausal women (10.6%) was similar to that reported in the general Finnish population of the capital region area in the 2000s (11%) [50]. For pre-menopausal women, the prevalence in our study, 21%, was markedly lower than that reported in the previously mentioned study (32%) and within the range of reported prevalence in the general European population: between 9.5% and 32% depending on country and exact age group [49,51]. ID_ferr_ rates in the Finnish blood donor population were reported in the 1980s [52] but the small sample size (around 50 frequent donors per sex-stratified group) and the use of different ferritin thresholds (12, 16.4 and 5.5 μg/L) make meaningful comparisons with the current data difficult. The reported 33% prevalence of ferritin < 16.7 μg/L for men is, however, markedly higher than the 6% prevalence in our population, suggesting that in men at least iron stores are better preserved than they were before. For women, our prevalence rates are lower than those reported in the Danish Blood Donor Study [1] (32%, 22% and 9% for pre-menopausal, post-menopausal and men, respectively) as well as than those from a recent Australian study (26.4% in females and 6.3% in males for WB donors) [2]. Iron deficiency rates reported in the RISE study cohort were higher but used different thresholds, making comparisons difficult [5].Differences in ID prevalence could result from the targeted iron supplementation policy, but also from other differences between study populations, such as minimal inter-donation interval, maximum number of annual donations or baseline population prevalence of iron deficiency. Overall, anemia and low hemoglobin rates were low in our donors.

The relationships between biomarkers and iron-deficiency thresholds were complex. We defined low ferritin using the WHO threshold of 15 μg/L [53] but high sTfR using the local University Hospital thresholds of 4.5 and 5 mg/L for women and men, respectively. As sTfR is thought to index iron-deficient erythropoiesis secondary to iron deficiency, one might expect donors with high sTfR to have low ferritin values [6]. However, whilst there was a visible negative relationship between ferritin and sTfR levels (Fig 1), 28% to 47% of ID donors had high sTfR but normal ferritin levels (ferritin ≥ 15 μg/L). Iron deficient erythropoiesis in blood donors could already be present at higher ferritin values [54,55], and indeed, raising the low ferritin threshold to 30 μg/L results in 95% to 97.5% of ID donors having both high sTfR and low ferritin. Furthermore, 80% of men with high sTfR also had ferritin < 30 μg/L, in line with a previous study [56]. These results seem to suggest that a ferritin threshold of around 30 μg/L could be more appropriate than the current WHO standard of 15 μg/L for screening donors at risk for iron-deficient erythropoiesis [54,57]. An important limitation to interpreting these results is the uncertainty regarding the assay-specific sTfR threshold which would appropriately identify iron-deficient erythropoiesis [58].

The relationship between iron deficiency and low hemoglobin and anemia in our blood donor population was not as straightforward as could be expected. Low hemoglobin and anemia in otherwise healthy blood donors are thought to result from iron deficiency, therefore, low hemoglobin and ID are expected to co-occur. In contrast, ferritin and sTfR levels were normal in 50% of anemic men and post-menopausal women and in 16% of anemic pre-menopausal women. Similarly, 34%, 49% and 69% of low hemoglobin pre-menopausal women, post-menopausal women and men, respectively, had normal ferritin and sTfR levels. Lack of sensitivity of the chosen sTfR or ferritin thresholds only partly explains these results, at least in men. For example, 27% of low Hb men had ferritin values over a less stringent 30 μg/L threshold (Fig 1). Overall these data underline the limits of using Hb screening as a tool to mitigate iron deficiency in blood donors.

Despite its promise as a biomarker indexing iron-deficient erythropoiesis [6], sTfR did not bring any added value to assessing donor’s iron status and its explanatory factors. Inter-individual variability in sTfR levels was only very partially explained by the variables available in the present study: at most the models explained 10% of the variance. Importantly, donation activity, which explained a large amount of inter-individual variability in ferritin levels, explained at most 6% of inter-individual variability in sTfR levels. STfR levels were therefore not as strongly affected by donation activity as ferritin levels, in line with previous studies reporting similar sTfR levels in new and frequent blood donors [14,56]. Similar to previous research suggesting that using a higher threshold for ferritin could be nearly as efficient as using an index combining sTfR and ferritin to identify IDE [57], our results provide evidence that sTfR does not necessarily significantly improve diagnosis of ID in blood donors.

Iron supplementation was not associated with higher iron levels in donors. Most men and post-menopausal women reported not being offered iron (65% and 57% respectively). Despite the FRCBS policy of offering iron to all women under 50 regardless of donation frequency, 35% of pre-menopausal women reported not being offered iron. Iron supplementation compliance was high: 60% to 65% of the donors offered iron reported taking all or almost all of the course and only 6% to 12% reported not ingesting any of the iron they were offered. Iron supplementation did not have any statistically significant effect on population ferritin levels in women and was associated with lower ferritin levels in men. Such results seem counter-intuitive given prior evidence of the positive effect of such supplementation on blood donor iron stores in different intervention studies [15,16,18,20,47]. Results from observational studies have been more contrasted. Self-administered iron supplementation was associated with a lower risk of iron deficiency [5] in a previous blood donor cohort study. In the Danish blood donor population, a negative relationship between iron supplementation provided by the blood establishment and ferritin levels was present, just as in ours [1]. Finnish donors are offered iron solely according to presumed menopausal status and recent donation activity. A negative relationship between iron supplementation and ferritin levels could therefore be explained by the non-randomized distribution of the treatment: donors who donate more frequently are both more likely to have reduced iron stores and more likely to be offered supplementary iron. The overall absence of a positive relationship between iron stores and supplementation could also result from insufficient amounts of iron being offered by the supplementation scheme. Finnish donors are provided with a short 20 to 30 day course of iron supplementation, with daily amounts provided varying between 9 and 20 mg of iron. While low daily doses of iron (19 mg) could be as effective as higher daily doses (38 mg) in mitigating iron loss in whole-blood donors [59], it may be that the length of the supplementation was simply too short. Indeed, even with iron supplementation it can take over 100 days for iron replete donors to recover 80% of iron stores or for iron deficient donors to recover to iron levels over 26 μg/L [15,60–62]. In light of these data, FRCBS may consider modifications to its iron supplementation policy to better accomplish its goal of mitigating iron deficiency in frequent blood donors. Additionally, despite similar results being obtained when taking account reported self-administered iron supplementation, our quantification of non-FRCBS iron intake is not precise enough for this study to be informative regarding such supplementation. Cohort studies on donor populations receiving larger amounts of iron and better controlling for self-administered iron supplementation will provide additional information regarding the effectiveness of such supplementation policies.

The most important predictors of inter-individual variability in ferritin levels were donation activity for all groups. The number of recent donations and time since last donation were negatively and positively, respectively, correlated with ferritin levels, consistent with previous studies [63,64]. The positive relationship between the square of the number of donations and ferritin levels indicates that the negative effect of increased donation frequency on ferritin levels was smaller for donors who donated more frequently than for donors who donated less frequently. Ferritin levels might reach a plateau in donors that are able to donate frequently without becoming anemic, as suggested by reports of lower prevalence of iron deficiency amongst the most frequent donors [2]. In contrast to physiologic and dietary factors such as BMI or red meat consumption which explained only 1–2% of inter-individual variance in ferritin levels, donation activity explained up to 27% of this variance. Time since last donation was the most consistent predictor of ferritin levels in all groups, even more so than donation activity in the previous two years. Shorter time since last donation has previously been associated with increased risk of iron deficiency [5] as well as lower ferritin levels [1], but our results highlight the large size when this effect is compared to other possible interventions. The very limited amount of variance explained by red meat consumption suggests that adequate dietary intake of iron could not by itself make up for high donation activity [65]. Similarly, the very small amount of variance attributed to iron supplementation suggests that it could not by itself compensate for donation-related blood loss.

Self-reported health of blood donors was good and was not correlated with ferritin levels, in line with results from the Danish Blood Donor study [29]. However, it remains unclear whether iron deficiency without anemia is associated with sub-optimal health outcomes in the general population [27,66,67]. Low ferritin in blood donors could still have subtle effects on donor health that would not be captured by a general self-reported health measure. Indeed, while self-rated health is considered to be the most feasible, inclusive, and informative measure of health status, it is non-specific and therefore the specific aspects of health that are emphasized in individual assessments cannot be controlled [68].

The present study provides an insight on the iron status of a blood donor population which is subject to an iron supplementation policy targeted towards at-risk groups. Targeted iron supplementation had only a limited effect on ferritin levels of blood donors: iron deficiency was less prevalent than in other donor populations but was nevertheless present in a non-negligible portion of the donor population. Furthermore, the population-level effect of donation activity dwarfed the effect of iron supplementation. Despite the fact that donor self-reported health was not affected by iron levels, further research is needed to identify an optimal, preferably individualized, donation interval as well as an optimal iron supplementation policy that ensures that blood donors are not subjected to any unnecessary risks related to iron depletion.

## Supporting information

S1 Fig Distribution of iron supplementation across study groups. Bars represent the number of donors for each possible response. The text above each bar provides the percentage over all donors.
(PNG)

S2 Fig Regression diagnostics for OLS regressions with ferritin as the outcome variable. The usual regression diagnostics for the OLS regressions with ferritin as the outcome show the presence of several outlier observations (colored in red).
(PNG)

S3 Fig Regression diagnostics for OLS regressions with sTfR as the outcome variable. The usual regression diagnostics for the OLS regressions with ferritin as the outcome show the presence of several outlier observations (colored in red).
(PNG)

S4 Fig Donor sTrR levels as a function of donation history, iron supplementation and red meat consumption. STfR levels are plotted as a function of number of donation in the last two years, days since last donation, iron supplementation and red meat consumption. Boxplots are superimposed for each number of donations, iron supplementation level and red-meat consumption level.
(PNG)

S5 Fig Ferritin levels as a function of age, BMI, beer consumption and wine consumption. Ferritin levels are plotted as a function of age, BMI, beer consumption and wine consumption. Boxplots are superimposed for each beer consumption and wine consumption level.
(PNG)

S6 Fig STfR levels as a function of age, smoking status, and milk consumption. STfR levels are plotted as a function of age, smoking status, and milk consumption. Boxplots are superimposed for each smoking status, and milk consumption level.
(PNG)

S7 Fig Distributions of self-reported health across study groups. The majority of donors report their health as being good or very good and only two percent rated their health as only average.
(PNG)

S1 Table Multivariable OLS regression analyses of ferritin levels. (PDF)

S2 Table Multivariable OLS regression analyses of sTfR levels. (PDF)

S3 Table Multivariable ordinal logistic regression analyses of self-reported health. (PDF)

## References

[1] Rigas AS, Sørensen CJ, Pedersen OB, Petersen MS, Thørner LW, Kotzé S, et al Predictors of iron levels in 14, 737 Danish blood donors: results from the Danish Blood Donor Study. Transfusion. 2014;54: 789–796. 10.1111/trf.12518 24372094 PMC4209803

[2] Salvin HE, Pasricha SR, Marks DC, Speedy J. Iron deficiency in blood donors: A national cross-sectional study. Transfusion. 2014;54: 2434–2444. 10.1111/trf.12647 24738792

[3] Goldman M, Uzicanin S, Osmond L, Scalia V, O’Brien SF. A large national study of ferritin testing in Canadian blood donors. Transfusion. 2017;57: 564–570. 10.1111/trf.13956 27943371

[4] Baart AM, Van Noord PAH, Vergouwe Y, Moons KGM, Swinkels DW, Wiegerinck ET, et al High prevalence of subclinical iron deficiency in whole blood donors not deferred for low hemoglobin. Transfusion. 2013;53: 1670–1677. 10.1111/j.1537-2995.2012.03956.x 23176175

[5] Cable RG, Glynn SA, Kiss JE, Mast AE, Steele WR, Murphy EL, et al Iron deficiency in blood donors: The REDS-II Donor Iron Status Evaluation (RISE) study. Transfusion. 2012;52: 702–711. 10.1111/j.1537-2995.2011.03401.x 22023513 PMC3618489

[6] Suominen P, Punnonen K, Rajamäki a, Irjala K. Serum transferrin receptor and transferrin receptor-ferritin index identify healthy subjects with subclinical iron deficits. Blood. 1998;92: 2934–9. Available: http://www.ncbi.nlm.nih.gov/pubmed/9763580 9763580

[7] Hastka J, Lasserre JJ, Schwarzbeck A, Reiter A, Hehlmann R. Laboratory tests of iron status: Correlation or common sense? Clin Chem. 1996;42: 718–724. 8653897

[8] Vuk T, Magnussen K, De Kort W, Folléa G, Liumbruno GM, Schennach H, et al International forum: An investigation of iron status in blood donors. Blood Transfus. 2017;15: 20–41. 10.2450/2016.0101-16 27643753 PMC5269425

[9] Rigas AS, Pedersen OB, Magnussen K, Erikstrup C, Ullum H. Iron deficiency among blood donors: Experience from the Danish Blood Donor Study and from the Copenhagen ferritin monitoring scheme. Transfusion Medicine. 2017 10.1111/tme.12477 29024114

[10] Kiss JE, Vassallo RR. How do we manage iron deficiency after blood donation? Br J Haematol. 2018;181: 590–603. 10.1111/bjh.15136 29767836

[11] Di Angelantonio E, Thompson SG, Kaptoge S, Moore C, Walker M, Armitage J, et al Efficiency and safety of varying the frequency of whole blood donation (INTERVAL): a randomised trial of 45 000 donors. Lancet. 2017;390: 2360–2371. 10.1016/S0140-6736(17)31928-1 28941948 PMC5714430

[12] O’Meara A, Infanti L, Stebler C, Ruesch M, Sigle JP, Stern M, et al The value of routine ferritin measurement in blood donors. Transfusion. 2011;51: 2183–2188. 10.1111/j.1537-2995.2011.03148.x 21517893

[13] Goldman M, Magnussen K, Gorlin J, Lozano M, Speedy J, Keller A, et al International Forum regarding practices related to donor haemoglobin and iron. Vox Sang. 2016;111: 449–455. 10.1111/vox.12431 27564140

[14] Mast AE, Bialkowski W, Bryant BJ, Wright DJ, Birch R, Kiss JE, et al A randomized, blinded, placebo-controlled trial of education and iron supplementation for mitigation of iron deficiency in regular blood donors. Transfusion. 2016;56: 1588–1597. 10.1111/trf.13469 26813849 PMC4905782

[15] Cable RG, Brambilla D, Glynn SA, Kleinman S, Mast AE, Spencer BR, et al Effect of iron supplementation on iron stores and total body iron after whole blood donation. Transfusion. 2016;56: 2005–2012. 10.1111/trf.13659 27232535 PMC4980154

[16] Marks DC, Speedy J, Robinson KL, Brama T, Capper HR, Mondy P, et al An 8-week course of 45 mg of carbonyl iron daily reduces iron deficiency in female whole blood donors aged 18 to 45 years: results of a prospective randomized controlled trial. Transfusion. 2014;54: 780–788. 10.1111/trf.12464 24660763

[17] Smith GA, Fisher SA, Doree C, Di Angelantonio E, Roberts DJ. Oral or parenteral iron supplementation to reduce deferral, iron deficiency and/or anaemia in blood donors. Cochrane Database Syst Rev. 2014;2014 10.1002/14651858.CD009532.pub2 24990381 PMC11019466

[18] Pasricha S-R, Marks DC, Salvin H, Brama T, Keller AJ, Pink J, et al Postdonation iron replacement for maintaining iron stores in female whole blood donors in routine donor practice: results of two feasibility studies in Australia. Transfusion. 2017;57: 1922–1929. 10.1111/trf.14173 28518220

[19] Magnussen K, Ladelund S. Handling low hemoglobin and iron deficiency in a blood donor population: 2 years’ experience. Transfusion. 2015;55: 2473–2478. 10.1111/trf.13152 25988343

[20] Bryant BJ, Yau YY, Arceo SM, Daniel-Johnson J, Hopkins JA, Leitman SF. Iron replacement therapy in the routine management of blood donors. Transfusion. 2012;52: 1566–1575. 10.1111/j.1537-2995.2011.03488.x 22211316 PMC3690467

[21] Cade JE, Moreton JA, O’Hara B, Greenwood DC, Moor J, Burley VJ, et al Diet and genetic factors associated with iron status in middle-aged women. Am J Clin Nutr. 2005;82: 813–820. 10.1093/ajcn/82.4.813 16210711

[22] Liu JM, Hankinson SE, Stampfer MJ, Rifai N, Willett WC, Ma J. Body iron stores and their determinants in healthy postmenopausal US women. Am J Clin Nutr. 2003;78: 1160–1167. 10.1093/ajcn/78.6.1160 14668279

[23] Fleming DJ, Jacques PF, Dallal GE, Tucker KL, Wilson PWF, Wood RJ. Dietary determinants of iron stores in a free-living elderly population: The framingham heart study. Am J Clin Nutr. 1998;67: 722–733. 10.1093/ajcn/67.4.722 9537620

[24] Jackson J, Williams R, McEvoy M, MacDonald-Wicks L, Patterson A. Is higher consumption of animal flesh foods associated with better iron status among adults in developed countries? A systematic review. Nutrients. 2016;8: 1–27. 10.3390/nu8020089 26891320 PMC4772052

[25] Galan P, Prezios P, Viteri F, Valeix P, Fieux B, Briancon S, et al Determining factors in the iron status of adult women in the SU.VI.MAX study. SUpplementation en VItamines et Mineraux AntioXydants. Eur J Clin Nutr. 1998;52: 383 Available: http://ovidsp.ovid.com/ovidweb.cgi?T=JS&PAGE=reference&D=cctr&NEWS=N&AN=CN-00687159 9683388 10.1038/sj.ejcn.1600561

[26] Fairweather-Tait SJ. Iron nutrition in the UK: getting the balance right. Proc Nutr Soc. 2004;63: 519–528. 10.1079/PNS2004394 15831123

[27] Pratt JJ, Khan KS. Non‐anaemic iron deficiency–a disease looking for recognition of diagnosis: a systematic review. Eur J Haematol. 2015;96: 618–628. 10.1111/ejh.12645 26256281

[28] Cook RL, Dwyer NJO, Parker HM, Donges CE, Cheng HL, Steinbeck KS, et al Iron Deficiency Anemia, Not Iron Deficiency, Young Women. 2017; 1–13. 10.3390/nu9111216

[29] Rigas AS, Pedersen OB, Sørensen CJ, Sørensen E, Kotz SR, Petersen MS, et al No association between iron status and self‐reported health‐related quality of life in 16,375 Danish blood donors: results from the Danish Blood Donor Study. Transfusion. 2015;55: 1–5. 10.1111/trf.12935 25851623

[30] Spencer BR, Kleinman S, Wright DJ, Glynn SA, Rye DB, Kiss JE. Restless legs syndrome, pica, and iron status in blood donors. Transfusion. 2013;53: 1645–1652. 10.1111/trf.12260 23763445 PMC4226336

[31] Partanen J, Niittymaki P, Nikiforow N, Palokangas E, Larjo A, Mattila P, et al FinDonor 10 000 study: A cohort to identify iron depletion and factors affecting it in Finnish blood donors. bioRxiv. Cold Spring Harbor Laboratory; 2018; 507665. 10.1101/507665 PMC7004091 31657023

[32] Team RDC, R Development Core Team R. R: A Language and Environment for Statistical Computing. R Found Stat Comput Vienna, Austria; 2016;1: 409 10.1007/978-3-540-74686-7

[33] WHO (World Health Organization). Serum ferritin concentrations for the assessment of iron status and iron deficiency in populations. Vitamin and Mineral Nutrition Information System. 2011; 1–5. Available: http://www.who.int/vmnis/indicators/serum_ferritin.pdf

[34] Kolbe-Busch S, Lotz J, Hafner G, Blanckaert NJC, Claeys G, Togni G, et al Multicenter evaluation of a fully mechanized soluble transferrin receptor assay on the Hitachi and Cobas Integra analyzers. The determination of reference ranges. Clin Chem Lab Med. 2002;40: 529–536. 10.1515/CCLM.2002.091 12113300

[35] Fox J. Robust regression. An R S-Plus companion to Appl Regres. 2002; 91.

[36] Rousseeuw PJ, Leroy AM. Robust regression and outlier detection John wiley & sons; 2005.

[37] Venables WN, Ripley BD. Modern Applied Statistics With S. Technometrics. Fourth. New York: Springer; 2003;45: 111–111. 10.1198/tech.2003.s33

[38] Maechler M. sfsmisc: Utilities from “Seminar fuer Statistik” ETH Zurich. [Internet]. 2018. Available: https://cran.r-project.org/package=sfsmisc

[39] Canty A, Ripley BD. boot: Bootstrap R (S-Plus) Functions. 2017.

[40] Efron B, Tibshirani RJ. An introduction to the bootstrap CRC press; 1994.

[41] Pasta DJ, others. Learning when to be discrete: continuous vs. categorical predictors SAS Global Forum 2009.

[42] Groemping U. Relative importance for linear regression in R: the package relaimpo. J Stat Softw. 2006;17: 139–147. 10.18637/jss.v017.i01

[43] De Bruin A, others. Health Interview Surveys: Towards International Harmonization of Methods and Instruments. WHO Regional Publications, European Series, No. 58. ERIC; 1996. 8857196

[44] Harrell Jr FE. rms: Regression Modeling Strategies [Internet]. 2018. Available: https://cran.r-project.org/package=rms

[45] Bürkner P-C. {brms}: An {R} Package for {Bayesian} Multilevel Models Using {Stan}. J Stat Softw. 2017;80: 1–28. 10.18637/jss.v080.i01

[46] Vehtari A, Gelman A, Gabry J. Practical Bayesian model evaluation using leave-one-out cross-validation and WAIC. Statistics and Computing. Springer US; 30 9 2016: 1–20. 10.1007/s11222-016-9696-4

[47] Gorlin J, Katz L, Elsmore D, Kirbach K, Erickson Y, Hove A, et al Prevalence of blood donor iron de fi ciency and feasibility ferritin-based iron replacement: a blood collection agency-based study. Vox Sang. 2016;49: 206–208. 10.1111/vox.12408 27172604

[48] WHO. the Global Prevalence of Anaemia in 2011. Document. 2015. 10.1017/S1368980008002401

[49] Fonseca C, Marques F, Robalo Nunes A, Belo A, Brilhante D, Cortez J. Prevalence of anaemia and iron deficiency in Portugal: The EMPIRE study. Intern Med J. 2016;46: 470–478. 10.1111/imj.13020 26841337

[50] Lahti-Koski M, Valsta LM, Alfthan G, Tapanainen H, Aro A. Iron status of adults in the capital area of Finland. Eur J Nutr. 2003;42: 287–292. 10.1007/s00394-003-0425-3 14564462

[51] Milman N, Taylor CL, Merkel J, Brannon PM. Iron status in pregnant women and women of reproductive age in Europe. Am J Clin Nutr. 2017;106: 1655S–1662S. 10.3945/ajcn.117.156000 29070543 PMC5701710

[52] Punnonen K, Rajamäki A. Evaluation of iron status of Finnish blood donors using serum transferrin receptor. Transfus Med. 1999;9: 131–134. 10.1046/j.1365-3148.1999.00191.x 10354382

[53] WHO. Serum ferritin concentrations for the assessment of iron status and iron deficiency in populations. [Internet]. Vitamin and Mineral Nutrition Information System. Geneva; 2011. (WHO/NMH/NHD/MNM/11.2)

[54] Dijkstra A, van den Hurk K, Bilo HJG, Slingerland RJ, Vos MJ. Repeat whole blood donors with a ferritin level of 30 μg/L or less show functional iron depletion. Transfusion. 2018; 1–5. 10.1111/trf.14433 30291758

[55] Hallberg L, Bengtsson C, Lapidus L, Lindstedt G, Lundberg P-A, Hultén L. Screening for iron deficiency: an analysis based on bone-marrow examinations and serum ferritin determinations in a population sample of women. Br J Haematol. Wiley Online Library; 1993;85: 787–798. 10.1111/j.1365-2141.1993.tb03225.x 7918045

[56] Flesland O, Eskelund AK, Flesland AB, Falch D, Solheim BG, Seghatchian J. Transferrin receptor in serum. A new tool in the diagnosis and prevention of iron deficiency in blood donors. Transfus Apher Sci. 2004;31: 11–16. 10.1016/j.transci.2004.01.011 15294189

[57] Kiss JE, Steele WR, Wright DJ, Mast AE, Carey PM, Murphy EL, et al Laboratory variables for assessing iron deficiency in REDS-II Iron Status Evaluation (RISE) blood donors. Transfusion. 2013;53: 2766–2775. 10.1111/trf.12209 23617531 PMC3895107

[58] Punnonen K, Irjala K, Rajamäki A. Serum transferrin receptor and its ratio to serum ferritin in the diagnosis of iron deficiency. Blood. 1997;89: 1052–7. Available: http://www.ncbi.nlm.nih.gov/pubmed/9028338 9028338

[59] Bialkowski W, Kiss JE, Wright DJ, Cable R, Birch R, D’Andrea P, et al Estimates of total body iron indicate 19 mg and 38 mg oral iron are equivalent for the mitigation of iron deficiency in individuals experiencing repeated phlebotomy. Am J Hematol. 2017;92: 851–857. 10.1002/ajh.24784 28494509 PMC5546996

[60] Kiss JE, Brambilla D, Glynn SA, Mast AE, Spencer BR, Stone M, et al Oral iron supplementation after blood donation: A randomized clinical trial. JAMA—J Am Med Assoc. 2015;313: 575–583. 10.1001/jama.2015.119 25668261 PMC5094173

[61] Schotten N, Jong PCMP, Moretti D, Zimmermann MB, Geurts-moespot AJ, Swinkels DW, et al The donation interval of 56 days requires extension to 180 days for whole blood donors to recover from changes in iron metabolism. Blood. 2016;128: 2185–2189. 10.1182/blood-2016-04-709451 27587880

[62] Niittymäki P, Arvas M, Larjo A, Mattila P, Ihalainen J, Syrjälä M, et al Retrospective analysis of capillary hemoglobin recovery in nearly 1 200 000 blood donor returns. Blood Adv. 2017;1: 961–967. 10.1182/bloodadvances.2016004218 The 29296737 PMC5737591

[63] Rigas AS, Pedersen OB, Erikstrup C, Hjalgrim H, Ullum H. Blood donation and iron deficiency. ISBT Sci Ser. 2017;12: 142–147. 10.1111/voxs.12309

[64] Peffer K, Den Heijer M, Holewijn S, De Graaf J, Swinkels DW, Verbeek ALM, et al The effect of frequent whole blood donation on ferritin, hepcidin, and subclinical atherosclerosis. Transfusion. 2013;53: 1468–1474. 10.1111/j.1537-2995.2012.03916.x 23043255

[65] Booth AO, Lim K, Capper H, Irving D, Fisher J, McNaughton SA, et al Iron status and dietary iron intake of female blood donors. Transfusion. 2014;54: 770–774. 10.1111/trf.12347 23876010

[66] Pasricha S-R, Low M, Thompson J, Farrell A, De-Regil L-M. Iron Supplementation Benefits Physical Performance in Women of Reproductive Age: A Systematic Review and Meta-Analysis. J Nutr. 2014;144: 906–914. 10.3945/jn.113.189589 24717371

[67] Houston BL, Hurrie D, Graham J, Perija B, Rimmer E, Rabbani R, et al Efficacy of iron supplementation on fatigue and physical capacity in non-anaemic iron-deficient adults: a systematic review of randomised controlled trials. BMJ Open. 2018;8: e019240 10.1136/bmjopen-2017-019240 29626044 PMC5892776

[68] Jylhä M. What is self-rated health and why does it predict mortality? Towards a unified conceptual model. Soc Sci Med. 2009;69: 307–316. 10.1016/j.socscimed.2009.05.013 19520474

