# Supplementary figures and images for: The effect of donation activity dwarfs the effect of lifestyle, diet and targeted iron supplementation on blood donor iron stores
Source: PLoS One. 2019 Aug 13;14(8):e0220862. doi: 10.1371/journal.pone.0220862 (PMC6692066; doi:10.1371/journal.pone.0220862)

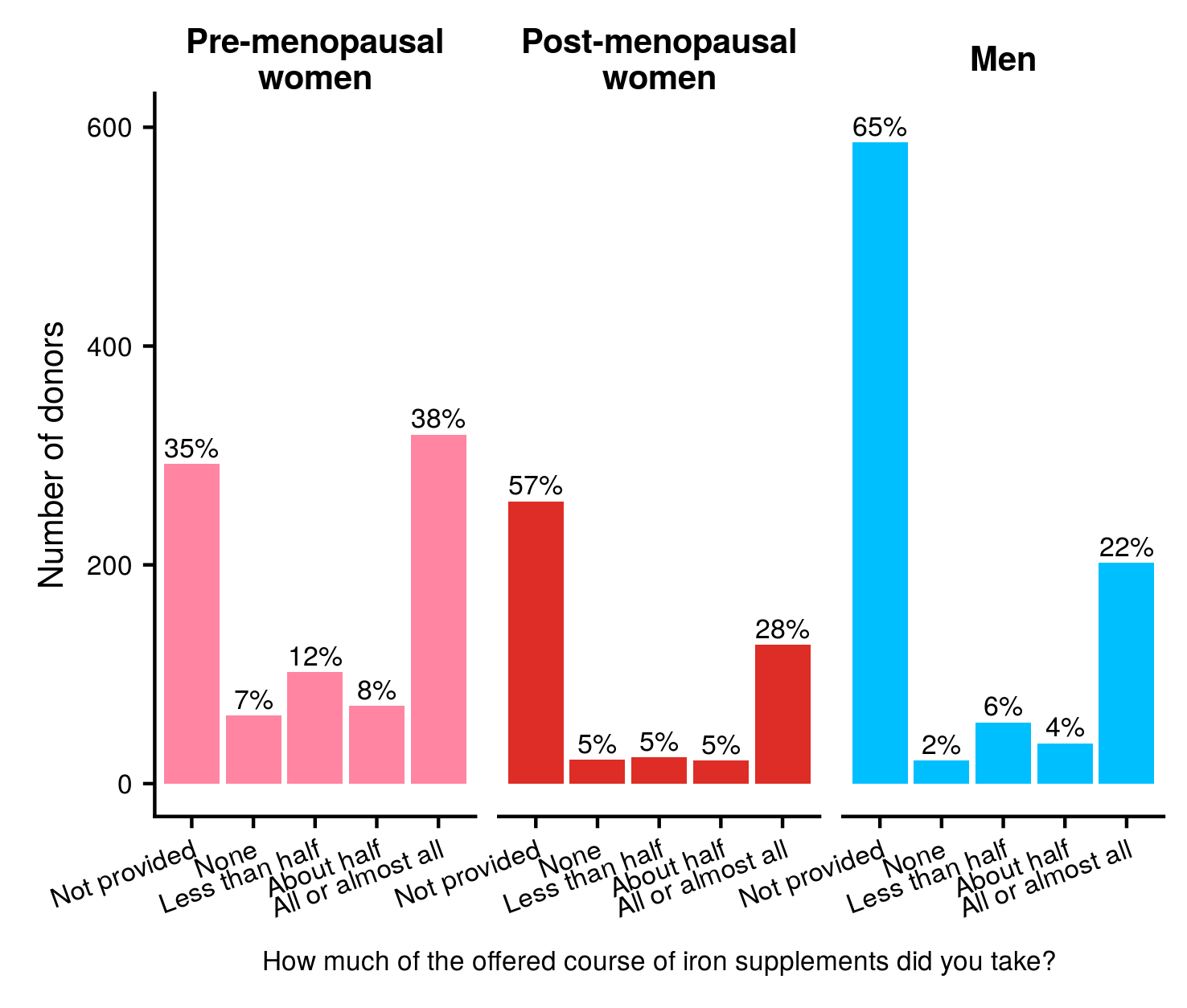

Supplement: S1 Fig — Bars represent the number of donors for each possible response. The text above each bar provides the percentage over all donors. (PNG) [file pone.0220862.s001.png]

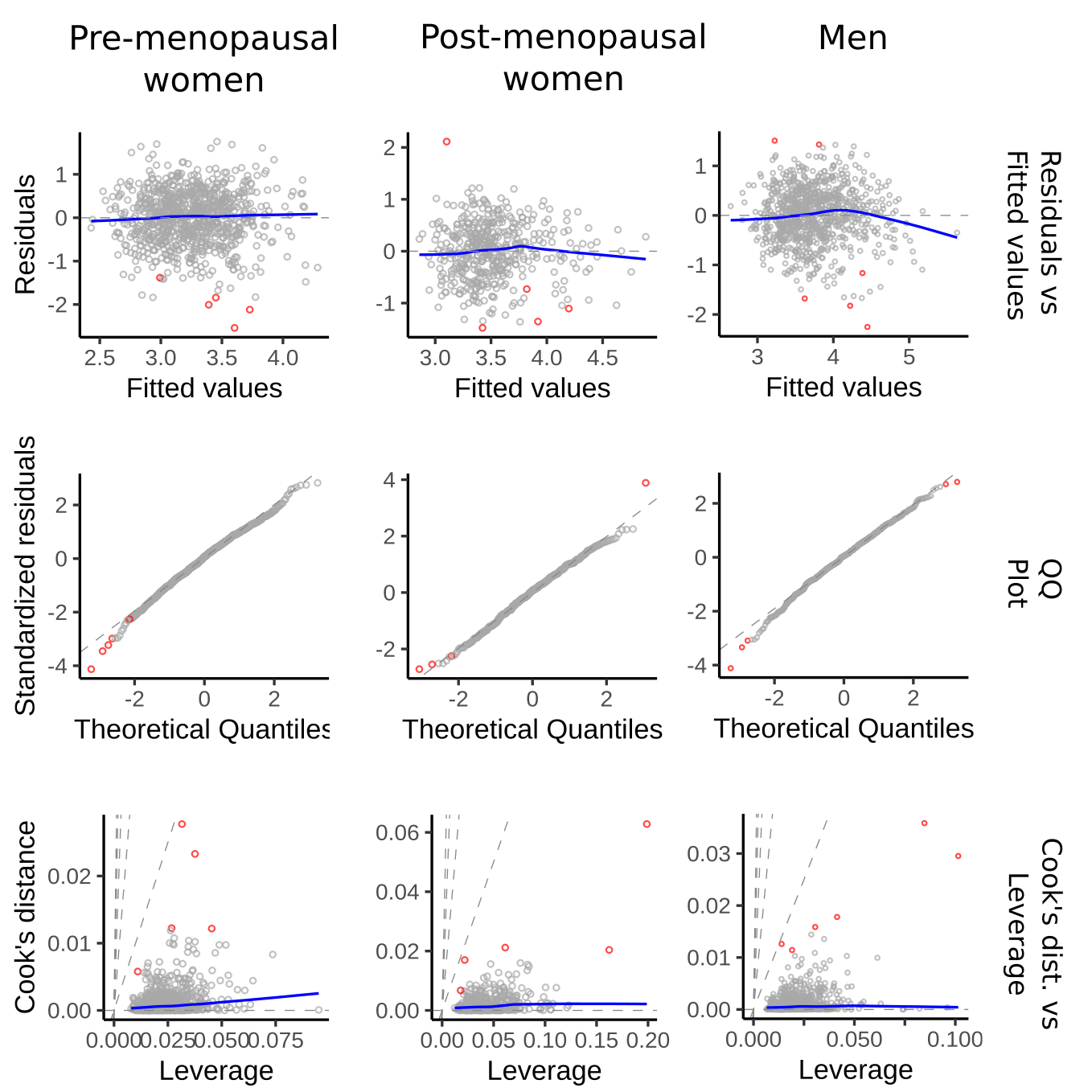

Supplement: S2 Fig — The usual regression diagnostics for the OLS regressions with ferritin as the outcome show the presence of several outlier observations (colored in red). (PNG) [file pone.0220862.s002.png]

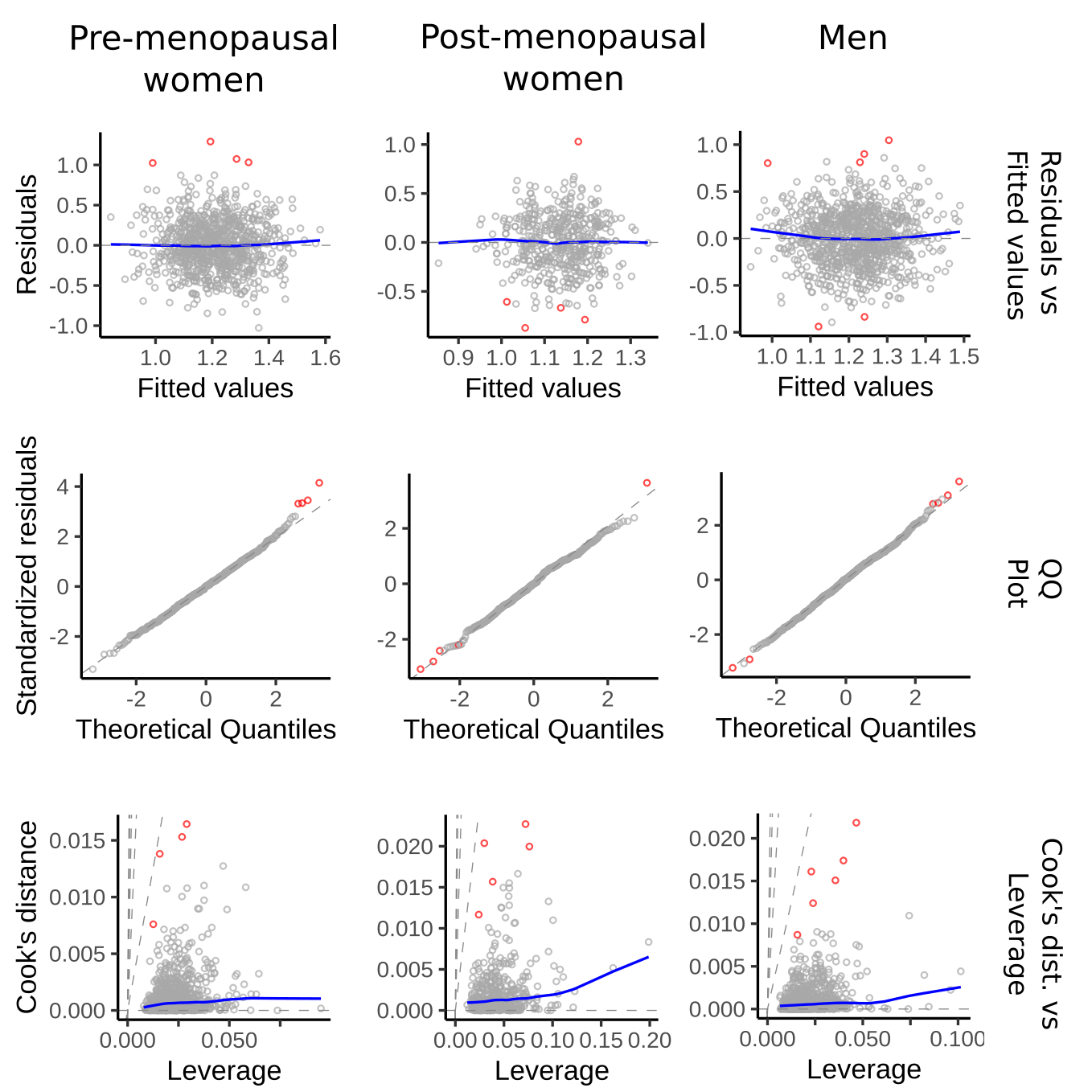

Supplement: S3 Fig — The usual regression diagnostics for the OLS regressions with ferritin as the outcome show the presence of several outlier observations (colored in red). (PNG) [file pone.0220862.s003.png]

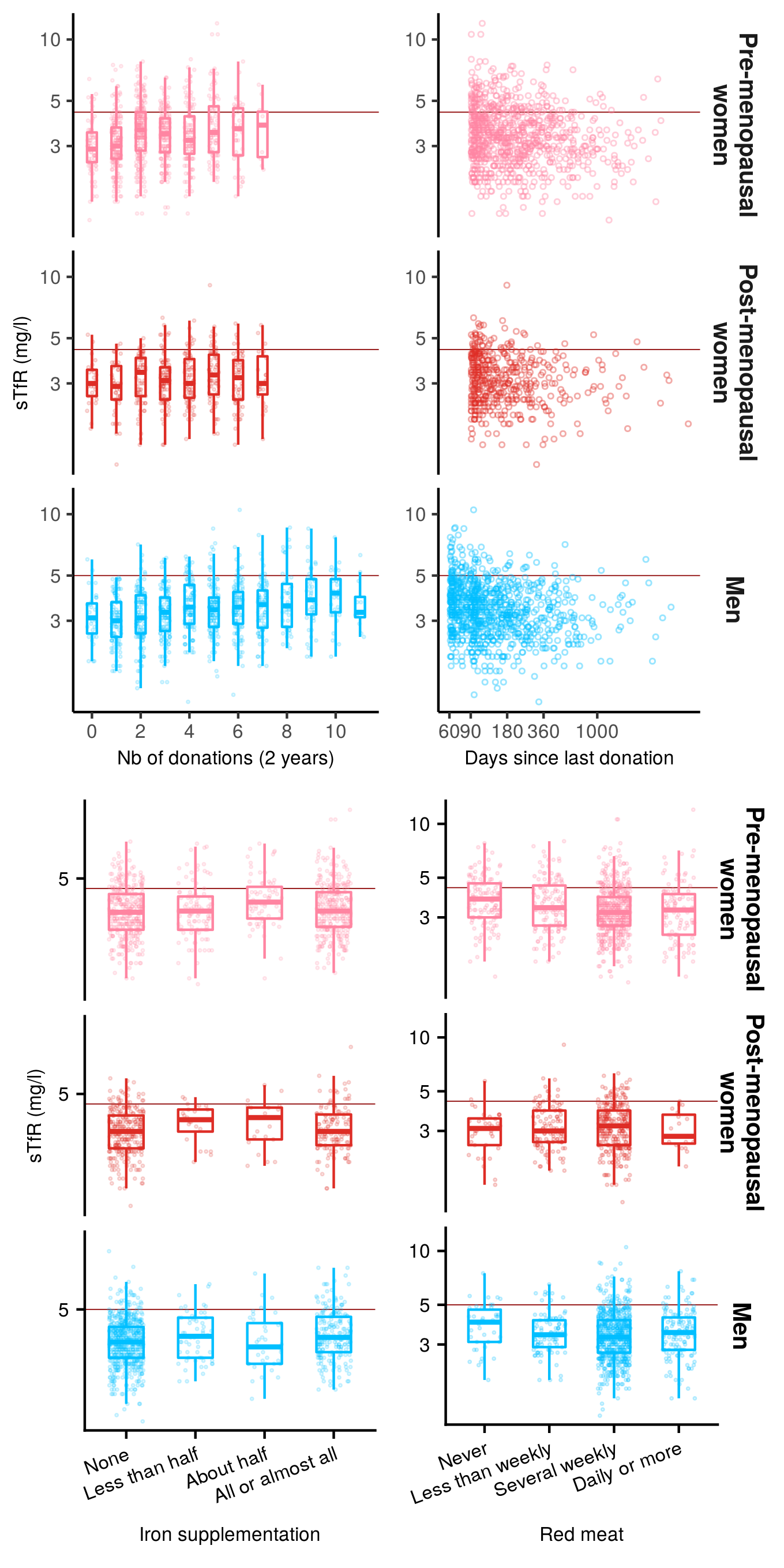

Supplement: S4 Fig — STfR levels are plotted as a function of number of donation in the last two years, days since last donation, iron supplementation and red meat consumption. Boxplots are superimposed for each number of donations, iron supplementation level and red-meat consumption level. (PNG) [file pone.0220862.s004.png]

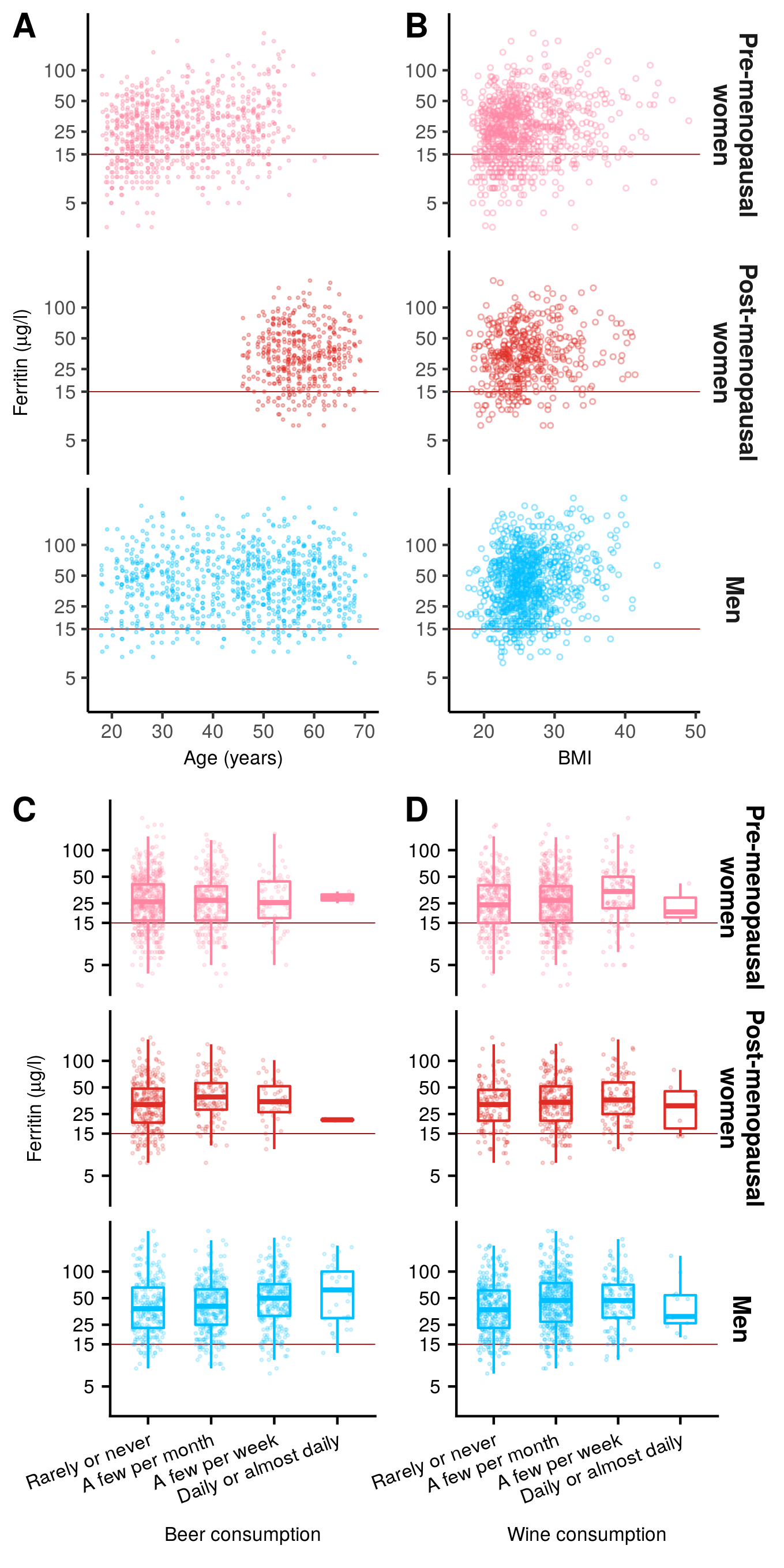

Supplement: S5 Fig — Ferritin levels are plotted as a function of age, BMI, beer consumption and wine consumption. Boxplots are superimposed for each beer consumption and wine consumption level. (PNG) [file pone.0220862.s005.png]

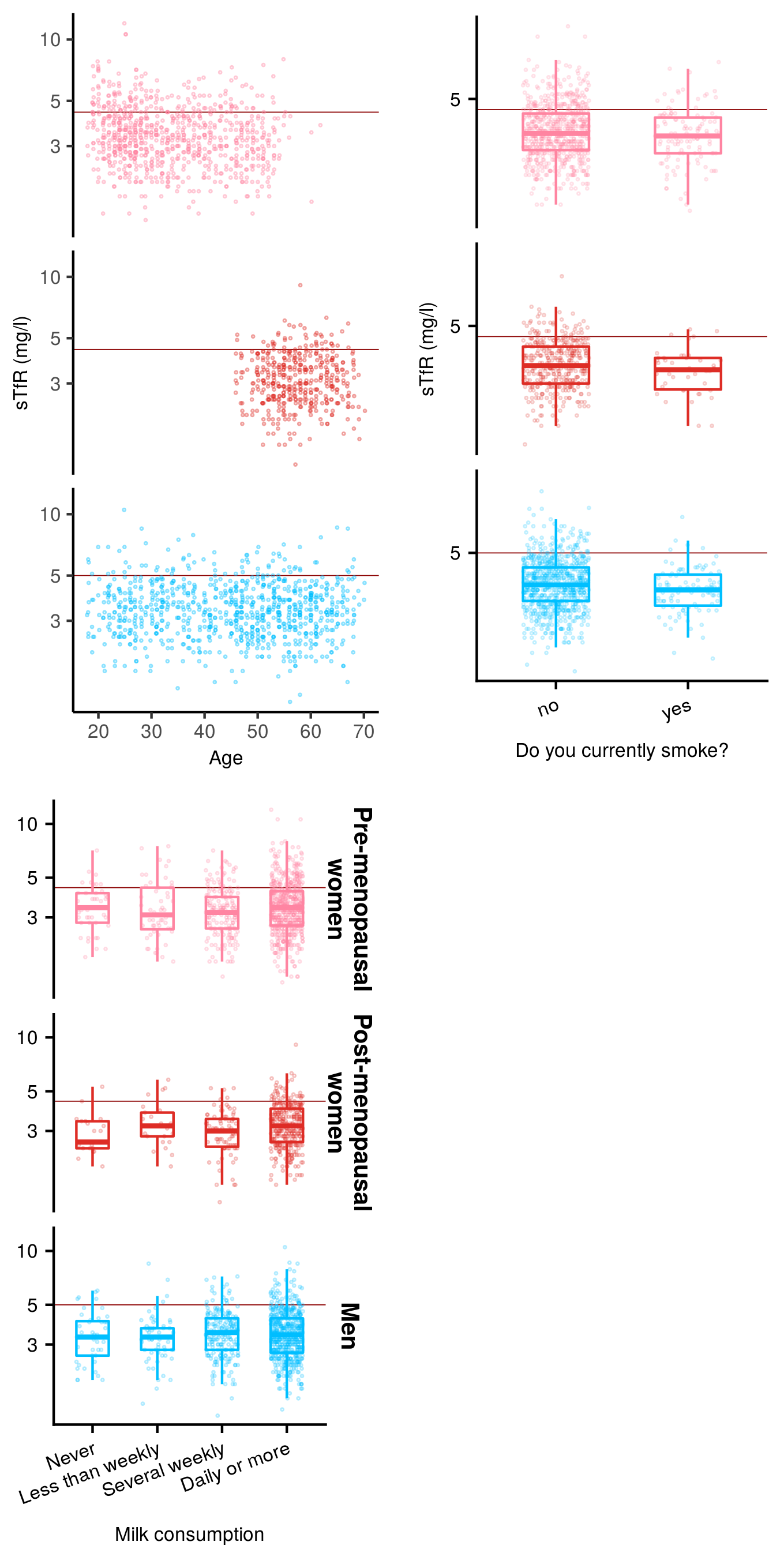

Supplement: S6 Fig — STfR levels are plotted as a function of age, smoking status, and milk consumption. Boxplots are superimposed for each smoking status, and milk consumption level. (PNG) [file pone.0220862.s006.png]

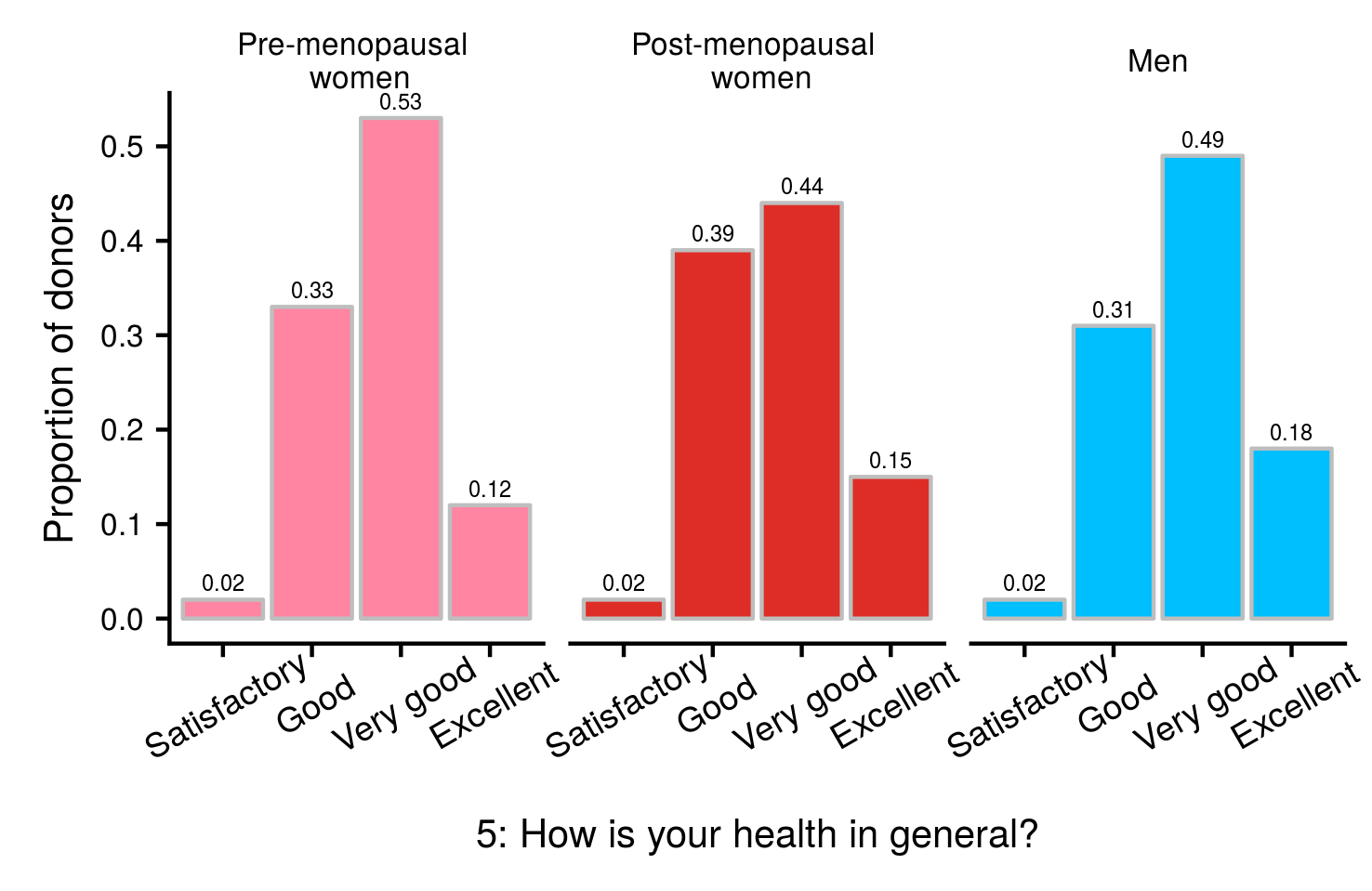

Supplement: S7 Fig — The majority of donors report their health as being good or very good and only two percent rated their health as only average. (PNG) [file pone.0220862.s007.png]
