# Supplementary material for: The effect of donation activity dwarfs the effect of lifestyle, diet and targeted iron supplementation on blood donor iron stores
Source: PLoS One. 2019 Aug 13;14(8):e0220862. doi: 10.1371/journal.pone.0220862 (PMC6692066; doi:10.1371/journal.pone.0220862)
Supplement: S1 Table — (PDF) [file pone.0220862.s008.pdf]

S1 Table. Multivariable OLS regression analyses of ferritin levels

	Pre-menopausal women		Post-menopausal women		Men	
	Coefficient (95% CI)	p-values	Coefficient (95% CI)	p-values	Coefficient (95% CI)	p-values
Age (5 years)	0.07 (0.04, 0.10)	< 0.0001	0.05 (0.01, 0.10)	0.038	0.02 (0.01, 0.04)	0.003
BMI	0.02 (0.01, 0.03)	0.002	0.02 (0.002, 0.03)	0.015	0.03 (0.02, 0.04)	< 0.0001
CRP	0.06 (-0.09, 0.21)	0.378	0.07 (-0.17, 0.28)	0.558	-0.01 (-0.23, 0.22)	0.933
Smoking(yes)	0.10 (0.02, 0.19)	0.030	0.07 (-0.06, 0.19)	0.310	0.08 (0.00, 0.16)	0.061
Pregnancy(Yes)	0.02 (-0.07, 0.11)	0.595	0.003 (-0.08, 0.08)	0.954		
Nb donations (2 years)	-0.06 (-0.09, -0.02)	0.003	-0.04 (-0.08, 0.00)	0.054	-0.09 (-0.11, -0.07)	< 0.0001
(Nb donations (2 years))²	0.01 (-0.005, 0.02)	0.184	0.01 (-0.01, 0.02)	0.313	0.01 (0.00, 0.01)	0.002
Time since last donation (days)	0.12 (0.07, 0.18)	< 0.0001	0.21 (0.14, 0.29)	< 0.0001	0.15 (0.11, 0.20)	< 0.0001
Iron supplementation	-0.003 (-0.03, 0.03)	0.866	-0.01 (-0.05, 0.03)	0.532	-0.05 (-0.08, -0.02)	0.003
Red meat	0.12 (0.06, 0.17)	< 0.0001	0.11 (0.03, 0.18)	0.008	0.10 (0.04, 0.16)	0.0005
Vegetables	-0.001 (-0.11, 0.11)	0.993	0.03 (-0.10, 0.17)	0.647	0.04 (-0.03, 0.11)	0.306
Fruit and Berries	0.02 (-0.07, 0.12)	0.598	-0.12 (-0.24, 0.01)	0.054	-0.01 (-0.07, 0.06)	0.867
Milk	-0.05 (-0.11, -0.002)	0.050	-0.03 (-0.10, 0.03)	0.317	-0.02 (-0.06, 0.03)	0.468
Fruit Juices	-0.01 (-0.07, 0.04)	0.602	0.004 (-0.05, 0.06)	0.890	-0.02 (-0.06, 0.02)	0.386
Coffee	0.02 (-0.01, 0.06)	0.237	-0.01 (-0.06, 0.05)	0.840	0.01 (-0.03, 0.05)	0.630
Tea	-0.02 (-0.06, 0.02)	0.320	0.04 (-0.01, 0.09)	0.111	0.01 (-0.02, 0.05)	0.454
Beer	0.002 (-0.07, 0.07)	0.963	0.08 (-0.0002, 0.16)	0.050	0.05 (0.01, 0.10)	0.031
Wine	0.05 (-0.02, 0.13)	0.150	0.08 (0.003, 0.15)	0.029	0.06 (-0.004, 0.11)	0.061
Liquor	0.01 (-0.09, 0.12)	0.811	-0.09 (-0.25, 0.05)	0.276	0.05 (-0.02, 0.12)	0.159
Observations	846		452		902	
R^2	0.20		0.27		0.37	
Adjusted R^2	0.18		0.23		0.36	
F Statistic	10.90*** (df = 19; 826)		8.28*** (df = 19; 432)		29.29*** (df = 18; 883))	

Note: ***p<0.01
