# Supplementary material for: The effect of donation activity dwarfs the effect of lifestyle, diet and targeted iron supplementation on blood donor iron stores
Source: PLoS One. 2019 Aug 13;14(8):e0220862. doi: 10.1371/journal.pone.0220862 (PMC6692066; doi:10.1371/journal.pone.0220862)
Supplement: S2 Table — (PDF) [file pone.0220862.s009.pdf]

S2 Table. Multivariable OLS regression analyses of sTfR levels

	Pre-menopausal women		Post-menopausal women		Men	
	Coefficient (95% CI)	p-values	Coefficient (95% CI)	p-values	Coefficient (95% CI)	p-values
Age (5 years)	-0.03 (-0.04, -0.01)	0.0004	-0.002 (-0.03, 0.02)	0.892	-0.01 (-0.02, -0.002)	0.016
BMI	0.003 (-0.001, 0.01)	0.207	0.001 (-0.005, 0.01)	0.728	0.004 (-0.001, 0.01)	0.128
CRP	0.03 (-0.03, 0.10)	0.389	0.04 (-0.07, 0.14)	0.488	0.12 (0.03, 0.21)	0.026
Smoking(yes)	-0.05 (-0.09, -0.005)	0.037	-0.07 (-0.14, -0.01)	0.045	-0.05 (-0.10, -0.01)	0.018
Pregnancy(Yes)	0.01 (-0.03, 0.06)	0.559	-0.03 (-0.08, 0.01)	0.153		
Nb donations (2 years)	0.02 (0.01, 0.04)	0.011	-0.002 (-0.02, 0.02)	0.851	0.02 (0.01, 0.03)	<0.0001
(Nb donations (2 years))^2	-0.004 (-0.01, 0.003)	0.241	0.001 (-0.01, 0.01)	0.865	-0.0002 (-0.003, 0.002)	0.888
Time since last donation (days)	-0.02 (-0.05, 0.004)	0.125	-0.04 (-0.08, -0.005)	0.029	-0.01 (-0.04, 0.01)	0.204
Iron supplementation	0.002 (-0.01, 0.02)	0.801	0.01 (-0.02, 0.03)	0.637	0.01 (-0.002, 0.03)	0.094
Red meat	-0.07 (-0.09, -0.04)	<0.0001	-0.0003 (-0.04, 0.04)	0.990	-0.03 (-0.06, -0.001)	0.048
Vegetables	-0.02 (-0.08, 0.04)	0.470	-0.001 (-0.07, 0.07)	0.984	-0.02 (-0.06, 0.03)	0.466
Fruit and Berries	0.01 (-0.04, 0.05)	0.694	0.01 (-0.06, 0.07)	0.871	-0.004 (-0.04, 0.03)	0.826
Milk	0.04 (0.01, 0.07)	0.004	0.03 (-0.003, 0.06)	0.073	0.02 (-0.01, 0.04)	0.218
Fruit Juices	-0.003 (-0.03, 0.02)	0.838	0.01 (-0.02, 0.03)	0.702	0.01 (-0.02, 0.03)	0.605
Coffee	-0.04 (-0.05, -0.02)	0.0005	-0.01 (-0.04, 0.02)	0.527	-0.01 (-0.03, 0.01)	0.317
Tea	0.01 (-0.01, 0.03)	0.511	-0.01 (-0.04, 0.02)	0.508	0.001 (-0.02, 0.02)	0.893
Beer	0.002 (-0.03, 0.04)	0.906	-0.02 (-0.06, 0.02)	0.289	-0.01 (-0.04, 0.01)	0.296
Wine	-0.01 (-0.04, 0.03)	0.621	-0.04 (-0.08, -0.01)	0.036	-0.03 (-0.06, -0.001)	0.056
Liquor	0.03 (-0.02, 0.09)	0.251	0.01 (-0.07, 0.09)	0.899	0.003 (-0.04, 0.04)	0.881
Observations	846		452		902	
R^2	0.12		0.06		0.09	
Adjusted R^2	0.10		0.02		0.08	
F Statistic	5.77*** (df = 19; 826)		1.42 (df = 19; 432)		5.06*** (df = 18; 883)	
