# Supplementary material for: The effect of donation activity dwarfs the effect of lifestyle, diet and targeted iron supplementation on blood donor iron stores
Source: PLoS One. 2019 Aug 13;14(8):e0220862. doi: 10.1371/journal.pone.0220862 (PMC6692066; doi:10.1371/journal.pone.0220862)
Supplement: S3 Table — (PDF) [file pone.0220862.s010.pdf]

S3 Table. Multivariable ordinal logistic regression analyses of self-reported health

	Women		Men	
	Odds-ratio (95% CI)	p-values	Odds-ratio (95% CI)	p-values
Health > Very Good	14.97 (8.40, 31.01)	< 0.0001	9.54 (5.00, 20.91)	< 0.0001
Health > Good	0.43 (0.25, 0.69)	0.001	0.38 (0.20, 0.69)	0.0005
Health > Satisfactory	0.03 (0.02, 0.06)	< 0.0001	0.04 (0.02, 0.06)	< 0.0001
Menopausal status	0.97 (0.67, 1.43)	0.866		
Age	0.95 (0.89, 1.02)	0.124	0.88 (0.83, 0.92)	< 0.0001
BMI	0.95 (0.93, 0.98)	0.0002	0.93 (0.90, 0.97)	0.0002
CRP	0.76 (0.52, 1.11)	0.150	1.08 (0.60, 2.17)	0.818
Smoking (yes)	0.84 (0.59, 1.18)	0.311	0.66 (0.43, 1.02)	0.053
Nb donations (2 years)	1.07 (0.99, 1.15)	0.092	1.08 (1.01, 1.15)	0.022
Time since last donation (days)	1.00 (0.89, 1.15)	0.940	1.02 (0.88, 1.17)	0.782
Daily physical activity	1.23 (1.07, 1.43)	0.005	1.26 (1.09, 1.48)	0.003
Exercise frequency	1.39 (1.22, 1.61)	< 0.0001	1.44 (1.25, 1.69)	< 0.0001
Ferritin (log)	1.11 (0.98, 1.25)	0.091	1.06 (0.91, 1.24)	0.468
Observations	1,298		902	
R^2	0.07		0.11	
chi^2	87.96^{***} (df = 10)		97.30^{***} (df = 9)	
